# Proxy-Based Diagnostics of Quantum Oracle Sketching Robustness for Non-IID Sensor and Telemetry Streams

**DOI:** 10.3390/s26175629

**Published:** 2026-09-04

**Authors:** Mohammed Farsi, Muhammad Mahmoud, Abdelmoniem Helmy

**Affiliations:** 1 Computer Information Systems Department, Taibah University, Yanbu 46477, Saudi Arabia; mafarsi@taibahu.edu.sa; 2 Information Systems Department, Faculty of Computers and Artificial Intelligence, Matrouh University, Matrouh 51511, Egypt; m.mahmoud@mau.edu.eg; 3 Department of Information Systems and Technology, Faculty of Graduate Studies for Statistical Research, Cairo University, Cairo 12613, Egypt

**Keywords:** sensor data streams, telemetry, streaming algorithms, non-IID data, correlation diagnostics, quantum computing, oracle sketching

## Abstract

Published quantum-streaming theory establishes quantum memory advantages for specific streaming problems; how a correlation-sensitive proxy model behaves under the structured dependence typical of sensor and telemetry streams, however, remains uncharted. We present a proxy-based diagnostic and hypothesis-generation framework for correlation-sensitive streaming analysis. Operational proxies for the refreshing time τ and repetition number *r*, introduced in this paper, are estimated on five synthetic non-IID regimes (IID, Markov switching, seasonal drift, burst repetition, and long-range dependence) and mapped onto a (τ,r) sensitivity landscape—the paper’s central artifact. Three memory-bounded classical baselines (online SGD, averaged SGD, and Count-Min) supply empirical reference points; no quantum algorithm is implemented or simulated and all quantum curves are heuristic proxy estimates. Analytic and empirical refreshing-time estimates diverge by up to 125× under long-range dependence (≈8× for Markov)—the two estimators answer different questions about temporal dependence. Using empirical τ, the proxy model predicts a hypothesised proxy-favourable region under mild-to-moderate Markov correlation that closes as correlation strengthens: the mean curves cross at $\rho^{*}\approx 0.82$ under the operating constants (C=2, δ=0.05; the crossing moves between $\rho^{*}\approx 0.38$ and beyond the sweep range across a *C*–δ grid, so only the ordinal reading is robust), and by ρ=0.88 the classical baseline exceeds the proxy estimate (Hodges–Lehmann difference 0.046). Applied unchanged to two real NAB telemetry streams, the same estimators place NYC Taxi inside and Machine Temperature outside the hypothesised favourable region—an ordering that persists across all tested encoder resolutions—with imbalance-aware metrics guarding against majority-class artefacts. Ablations over the τ estimator, forward window, stream length, target function, and proxy constants preserve the regime ordering within each estimator family.

## 1. Introduction

The exponential growth of data-intensive applications in domains such as sensor networks, financial markets, climate monitoring, and telecommunications has created an urgent need for streaming algorithms that process data on the fly with severely constrained memory [1]. Classical streaming algorithms—including sketching methods, online learners, and sliding-window estimators—offer practical solutions but face fundamental memory–accuracy tradeoffs rooted in information-theoretic lower bounds [2]. A further complication is that real-world streams are rarely IID: Internet traffic exhibits long-range dependence and self-similarity [3,4], and streaming-ML systems must contend with concept drift and non-stationary input distributions [5,6,7,8].

Published quantum-streaming results establish quantum space advantages for specific natural streaming problems, including an exponential separation for approximating maximum directed cut [9,10,11]. Motivated by this, we use *quantum oracle sketching* as a manuscript-level label for the proxy-based diagnostic introduced below; no published source is claimed to establish this paper’s operational proxies or their performance. Such quantum advantages are problem- and access-model-specific, not universal empirical findings [12,13,14]. The broader quantum ML landscape includes quantum linear systems [15], quantum feature maps and data encoding [16,17], general quantum-ML perspectives [18,19,20,21], and classical shadow tomography [22].

A particularly notable challenge for this diagnostic is non-IID data. Temporal dependence and exact duplication can reduce the effective information available in a stream; this paper summarises those effects through the operational quantities *refreshing time* and *repetition number*. Their behaviour under structured, real-world correlation patterns motivates the present study.

Four questions motivate our study: (RQ1) How tight are our generic analytic proxies, relative to their empirical counterparts, for specific structured families? (RQ2) How informative are τ and *r* as effective-sample-budget summaries? (RQ3) Under what correlation conditions does the proxy-estimated favourable separation persist, degrade, or potentially improve? (RQ4) How do empirically measured (τ,r) relate to the analytic proxy predictions?

### Scope

The scope is for a **proxy-based computational study** of data-processing viability for sensor-style streams: operational correlation proxies introduced in this paper are used to map where proxy-estimated performance is most favourable under the modelling assumptions across structured synthetic non-IID conditions of the kinds documented for sensor and telemetry traffic (Markov switching, seasonal cycles, burst repetition, and long-range dependence), with a two-dataset real-stream sanity check on NAB telemetry streams—an urban demand series and a machine-temperature sensor series (Section 6.8). Quantum performance curves are heuristic proxies and no quantum algorithm is implemented; circuit simulation, formal analysis, and broader real-stream validation remain explicit follow-on targets (Section 9). The paper is therefore best read as a **diagnostic and hypothesis-generation framework** for correlation-sensitive streaming analysis: it produces falsifiable, proxy-level predictions about where quantum oracle sketching is most likely to remain robust, and it neither demonstrates nor claims a realised quantum–classical separation.

We study five non-IID regimes (IID, Markov switching, seasonal drift, burst repetition, and long-range dependence), specifying analytic proxy expressions for τ and *r*, measuring their empirical counterparts on generated streams, comparing three classical baselines, and mapping results onto a (τ,r) sensitivity landscape with 10-seed means, 95% CI bands, and paired comparisons interpreted through effect sizes and interval estimates.

Our principal contributions are as follows:

1. **Operational correlation proxies** for five structured non-IID regimes, explicitly labeled as engineering approximations rather than theorems.
2. **Empirical-vs-proxy gap analysis** showing that, where analytic proxies overestimate empirical refreshing time, the gap ranges from roughly 8× (Markov) to 125× (long memory); in the IID and burst regimes the empirical values instead exceed the analytic proxy, reflecting finite-sample autocorrelation fluctuations and, for burst, forward-window counting effects at the chosen window length. These directional gaps indicate where tighter family-specific characterisations are most needed.
3. **A (τ,r) sensitivity landscape** that maps the quantum accuracy proxy as a function of correlation parameters, identifying regions where the proxy accuracy is highest; this landscape is the paper’s central diagnostic and hypothesis-generation artifact.
4. **Regime-dependent robustness hypotheses:** the proxy model predicts a hypothesised proxy-favourable region under mild to moderate correlation that narrows predictably as correlation strengthens. These are proxy-level hypotheses intended for future algorithm-level validation, not demonstrated quantum performance.

Section 2 and Section 3 review related work and introduce the proxy model; Section 4 specifies the correlation proxies; Section 5 describes the experimental setup; Section 6 presents nine experiments (seven on synthetic streams, a two-dataset real-stream sanity check, and an ablation suite); Section 7 discusses implications, the analytic–empirical divergence, and the scope of the diagnostic; Section 8 systematises threats to validity; Section 9 outlines future work; and Section 10 concludes.

## 2. Related Work

### 2.1. Classical Streaming Algorithms

Classical data streaming has been studied extensively since the seminal work of Alon et al. [2] on frequency moments [1]. Key developments relevant to this paper include the Count-Min Sketch of Cormode and Muthukrishnan [23] for frequency estimation, the Frequent Directions algorithm of Ghashami et al. [24] for streaming low-rank approximation, and the sketching-as-linear-algebra framework of Woodruff [25]. On the learning side, Shalev-Shwartz [26] provides the standard reference for online convex optimisation; Polyak and Juditsky [27] introduced Polyak–Ruppert averaging, which we use as a second classical baseline; and Weinberger et al. [28] established the feature-hashing technique underlying our bounded-memory classifier.

The behaviour of streaming algorithms under structured temporal correlation has received comparatively less attention. Concept drift is surveyed by Gama et al. [6] and Ditzler et al. [29]; ADWIN [30] is the canonical adaptive-window solution. Broader online-learning treatments appear in MOA [31], Hoi et al. [32], and Gama [33]; the most recent systematic drift review is Arora et al. [5]; adaptive random forests [34] serve as the drift-aware classical reference. Recent work on non-stationary streaming includes elastic online deep-learning pipelines [7], bi-dynamic-distribution learning [8], and evolving-prototype classifiers for imbalanced streams [35]. Non-stationary online optimisation is analysed in Besbes et al. [36] and Ben-David et al. [37]; Count-Min refinements appear in Ben Mazziane et al. [38] and Cormode et al. [39]; and foundational self-similarity measurements are in Park and Willinger [40].

### 2.2. Quantum Advantages for Data Processing

Quantum algorithms for data processing include quantum SVMs [41], quantum PCA [42], and the quantum singular value transformation [43] with block-encoding subroutines [44]. Critical perspectives on access-model dependencies appear in Aaronson [12] and Tang [14]; qRAM feasibility is discussed in Giovannetti et al. [45]; surveys include Chen et al. [46] and Schuld and Petruccione [21]. Quantum advantages depend critically on data structure and access [13]; an experimental demonstration of exponential advantage appears in Huang et al. [47]. On quantum streaming, Kallaugher [9] established the first quantum space advantage for a natural streaming problem, Kallaugher et al. [11] strengthened this to an exponential quantum-classical space separation, and Kallaugher et al. [10] provided a black-box quantum-sketch construction for classical algorithm designers.

### 2.3. Non-IID Data in Quantum Computing

Arunachalam and de Wolf [48] study quantum sample complexity under the IID PAC assumption. The effect of noisy, correlated, and time-varying classical streams on quantum-streaming diagnostics remains comparatively underexplored. We introduce paper-defined operational proxies across five structured regimes; the Markov mixing time follows product-chain results [49] and the long-memory statistics follow fGn theory [50,51].

## 3. Preliminaries

### 3.1. Quantum Oracle Sketching

In this paper, *quantum oracle sketching* denotes the proxy-based diagnostic framework studied here: published quantum-streaming and sketching results provide general motivation [9,10,11], while the correlation quantities and performance proxy are operational constructs introduced in this paper. We use the terms *refreshing time* for a decorrelation-scale diagnostic and *repetition number* for an expected-duplication diagnostic, with the precise definitions given in Section 3.2. This nomenclature does not attribute the proxy equations, memory-reference curves, or correlation guarantees to the cited quantum-streaming papers.

### 3.2. Heuristic Effective-Sample Model

**Proxy status.** The quantities τ and *r* defined below are *paper-defined operational proxies*; Section 3.3 separates published background from this paper’s assumptions and heuristic constructions. We introduce a simplified effective-sample proxy for computational exploration across diverse correlation families. The choice C=2, δ=0.05 in Equation (2) rescales accuracy contours uniformly without deforming the qualitative regime ordering: changing *C* or δ shifts every contour along the hyperbolic family $\tau r\propto {\left(C^{2}\log\left(1/\delta \right)\right)}^{-1}$, preserving the relative ranking of all regimes—although the *absolute* location of any proxy–baseline crossover does shift with these constants, as quantified in Section 6.9 (Table 8). Practitioners interpreting the landscape quantitatively should treat absolute contour values as indicative rather than universal.

**Definition** **1** (Refreshing Time—operational proxy)**.** *We define the* operational refreshing time *τ of a data stream as the smallest lag at which the normalized autocorrelation drops below 1/e. This is an empirically measurable quantity that serves as a proxy for the decorrelation rate of the stream.*

**Definition** **2** (Repetition Number—operational proxy)**.** *We define the* operational repetition number *r as 1 plus the expected number of* exact duplicate *copies of each sample within a forward window of length W. Throughout this paper we use W=20 in the empirical estimator; this is the definition that Figure 4b reports and matches to the analytic family-level r in units.*

We model the effective independent sample count as

(1) $$T_{\mathrm{eff}}=T/\left(\tau \cdot r\right),$$

and use the following heuristic oracle error proxy:

(2) $$\epsilon \leq C\sqrt{\frac{n\log(1/\delta )}{T_{\mathrm{eff}}}},$$

where C=2 and δ=0.05 throughout our experiments. Under IID data, τ=1 and r=1, recovering the standard scaling $\epsilon ∼\sqrt{n/T}$.

The heuristic classification accuracy proxy used in our experiments is

(3) $$\mathrm{acc}\_\mathrm{proxy}=\max\;\left(0.5,\;1-\epsilon^{2}\right),$$

where the floor of 0.5 represents the random-guess baseline for binary classification. When $\epsilon^{2}>0.5$ (as occurs in the long-memory regime), the proxy saturates at 0.5, reflecting that the model predicts no useful signal. This is an engineering surrogate that translates oracle approximation quality into a classification metric for visualization.

### 3.3. Relationship Between Operational Proxies and Quantum Oracle-Sketching Theory

This subsection separates the present study’s published background, assumptions, and heuristic constructions; Table 1 summarises the provenance and epistemic status of every quantity used in the paper.

#### 3.3.1. Published Theory and Boundary

Published quantum-streaming theory establishes a polynomial quantum space advantage for one-pass triangle counting [9], an exponential quantum space advantage for approximating maximum directed cut [11], and a black-box route from certain classical sketches to quantum streaming algorithms [10]. These are problem-specific results; they do not establish the operational τ, *r*, $T_{\mathrm{eff}}$, or accuracy model used here. This paper adds no quantum theorem. The τ-penalised landscape studied here is a *classical effective-sample-size stress test* used for diagnostic and hypothesis-generation purposes, not a rendering of a published quantum cost theorem.

#### 3.3.2. Assumptions

We assume (a) that a stream’s temporal dependence can be usefully summarised by two scalars—a decorrelation scale and a duplication rate; (b) that binarised feature streams (Section 5) retain enough of the correlation structure of the underlying process for such scalars to be meaningful; and (c) that a task-level statistical generalisation rate evaluated at an effective sample count is a useful stand-in for end-to-end pipeline behaviour. These are the modelling choices of this paper.

#### 3.3.3. Heuristic Constructions Introduced in This Paper

Equations (1) and (2) are paper-defined constructions rather than published quantum guarantees. First, Equation (1) divides by both τ and *r*, using a classical effective-sample-size correction ($T_{\mathrm{eff}}\approx T/\tau_{\mathrm{int}}$ in the sense long standard in Markov-chain output analysis [52]) and an additional duplicate-count penalty. Our $T_{\mathrm{eff}}$ should therefore be read as a pessimistic effective-information budget, not as a prediction of a quantum sample-complexity theorem. Second, the $\sqrt{n/T_{\mathrm{eff}}}$ template in Equation (2) is the statistical generalisation rate for the *n*-dimensional linear classification task studied here (VC dimension n+1) applied to the effective sample count; it is *not* the sample cost of an implemented quantum procedure. Throughout, *oracle error* accordingly denotes the approximation error of our heuristic model’s decision rule, not a physical or algorithmic oracle-construction error (see Experiment 6).

#### 3.3.4. Memory References

Published quantum-streaming papers establish problem-specific quantum space advantages [9,11]; they do not establish the O(n) and $\Omega (\sqrt{N})$ pair for the diagnostic task studied here. We retain that pair solely as a paper-defined *illustrative scaling reference*: O(n) represents a linear-in-feature quantum memory curve and $\Omega (\sqrt{N})$ a sublinear-in-alphabet classical curve. Neither is an empirically witnessed floor or ceiling for any learner studied here: no experiment measures a memory lower bound for online SGD, averaged SGD, or Count-Min, and for the 10-dimensional linear classification task used in our experiments the classical learners are sample-limited rather than memory-limited once *M* exceeds a small threshold (Section 6.2). The curves therefore visualise a hypothetical asymptotic contrast, not an established separation for this task.

##### Scope Limitations

The operational τ and *r* are measurement conventions, not theoretical quantities: they are computable from any stream but are not formally equivalent to a universal mixing time, effective-sample count, or quantum sample-overhead parameter. As Section 7.3 shows, a single scalar can diverge substantially across reasonable dependence summaries. Consequently, positions on the (τ,r) landscape are diagnostic coordinates, and every “favourable”/“unfavourable” designation in this paper is a statement about the proxy model evaluated at those coordinates, not about any realised quantum computation. Establishing formal foundations or bounding the proxy error is follow-on theoretical work (Section 9).

## 4. Correlation Proxies for Specific Data Families

We specify heuristic expressions for the operational τ and *r* under five concrete correlation regimes. Each is an engineering proxy motivated by the autocorrelation or mixing structure of the regime; formal bounds on its relation to effective information are for follow-on work.

### 4.1. Regime 1: IID Baseline

Under IID sampling from a uniform distribution over ${\left\{0,1\right\}}^{n}$

(4) $$\tau_{\mathrm{IID}}=1,\;r_{\mathrm{IID}}=1,\;T_{\mathrm{eff}}=T.$$

This serves as the reference against which all other regimes are compared.

### 4.2. Regime 2: Markov Switching

Consider a Markov chain on ${\left\{0,1\right\}}^{n}$ where each bit flips independently with probability (1−ρ)/2 at each step for correlation parameter ρ∈[0,1). The stationary distribution is uniform.

The mixing time of this product chain is Θ(nlogn/(1−ρ)). We use the simplified engineering proxy

(5) $$\tau_{\mathrm{Markov}}\left(\rho ,n\right)\approx \frac{n}{1-\rho },$$

which preserves the dominant divergence as ρ→1 while suppressing logarithmic and model-specific constants. *Provenance:* the 1/(1−ρ) scaling is theory-motivated (mixing-time asymptotics), the omission of the logn factor is an engineering simplification, and its use as an effective-information penalty is unvalidated. Since samples from a Markov chain are not repeated exactly, $r_{\mathrm{Markov}}=1$, giving $T_{\mathrm{eff}}\approx T\left(1-\rho \right)/n$.

### 4.3. Regime 3: Seasonal Drift

Consider a stream where bit *i* at time *t* is drawn as Bernoulli with probability $p_{i}\left(t\right)=\frac{1}{2}+\alpha \sin\left(2\pi t/P+\phi_{i}\right)$, where *P* is the period, α∈(0,0.5) is the drift strength, and $\phi_{i}$ are fixed phase offsets.

The seasonal regime is *distributional drift*: samples at distinct times are conditionally independent given *t*, making this non-IID only in the weak sense of non-stationary marginals. It is included because both the proxy diagnostic and classical learners are affected by marginal drift, and because it is a common practical stream pattern.

We propose the decorrelation proxy

(6) $$\tau_{\mathrm{Seasonal}}\left(P,\alpha \right)=\max\left(1,\;P\cdot \alpha \right),$$

lower-bounded by 1 to ensure $\tau \geq \tau_{\mathrm{IID}}$ in all cases. *Provenance:* the product Pα captures the intuition that decorrelation scales with both period and drift strength; the functional form max(1,Pα) is an engineering convenience with no formal derivation. Since the distribution changes continuously, $r_{\mathrm{Seasonal}}=1$.

### 4.4. Regime 4: Burst Repetition

In the burst model, at each step, with probability $p_{b}$ the previous sample is exactly repeated for an additional *L* consecutive copies (i.e., the burst contributes *L* duplicate observations). The expected number of observations per effective fresh sample is therefore

(7) $$r_{\mathrm{Burst}}\left(L,p_{b}\right)=1+p_{b}\cdot L.$$

*Provenance:* the formula follows directly from the model definition (theory-motivated); the assumption that duplicates contribute no new information is exact for our synthetic generator but would require validation on real burst-prone streams. Since bursts do not affect the mixing of fresh samples, $\tau_{\mathrm{Burst}}=1$, giving $T_{\mathrm{eff}}=T/\left(1+p_{b}L\right)$.

### 4.5. Regime 5: Long-Range Dependence

For streams driven by fractional Gaussian noise (fGn) with Hurst parameter H∈(0.5,1), the autocorrelation of the *underlying Gaussian process* decays as a power law and integrated autocorrelation grows as $T^{2H-1}$. We adopt as our proxy the horizon-dependent correlation penalty

(8) $$\tau_{\mathrm{LRD}}\left(H,T\right)=T^{2H-1},$$

yielding $T_{\mathrm{eff}}=T^{2(1-H)}$.

#### Proxy Status

Equation (8) is an *upper-bound proxy*. In our construction the stream is a *binarized* fGn (each bit is a thresholded fGn path); binarization converts Gaussian autocorrelation γ(k) into binary autocorrelation (2/π)arcsin(γ(k)) by the arcsine law for clipped Gaussian signals [53], which is strictly smaller in absolute value. The effective decorrelation of the binary stream is therefore *faster* than Equation (8) suggests, so the proxy is conservative. Deriving a tighter proxy that accounts for the arcsine transformation is deferred to future work. Unlike the other regimes, this proxy depends on the stream length *T*, reflecting the property that the underlying driving process has no finite decorrelation time.

### 4.6. Summary

Each regime idealises a documented stream phenomenon: Markov switching captures short-range state persistence in sensor readouts; seasonal drift the diurnal and weekly cycles of telemetry and demand series [5,6]; burst repetition the duplicate-heavy event bursts of monitoring and logging pipelines; and long-range dependence the self-similar traffic behaviour first measured on Ethernet [3,4]. They are idealisations, not replicas: real streams mix these mechanisms (as the two NAB cases of Section 6.8 show), and the mapping from regime parameters to any concrete deployment is qualitative (Table 10).

Table 2 summarises the proxy parameters and heuristic MSE proxy values for each regime, computed from the single master formula $\epsilon^{2}=\min\left(1,\;C^{2}n\log\left(1/\delta \right)/T_{\mathrm{eff}}\right)$ with C=2 and δ=0.05.

## 5. Experimental Framework

Throughout this section, the comparison is between (1) empirical bounded-memory classical learners evaluated on a simple linear classification task, and (2) a literature-motivated heuristic proxy for quantum oracle sketching performance. The empirical classical baselines are not instances of the problem-specific separations in the published quantum-streaming literature.

### 5.1. Data Generation and Diagnostics

Data streams over ${\left\{0,1\right\}}^{n}$ with n=10 (N=1024) and T≤20,000. Target task: binary classification with random linear separator $f\left(x\right)=1[\langle x-\frac{1}{2}1,w\rangle >0]$, $w\in R^{n}$ fixed. Parameter values follow Section 4: IID (uniform), Markov (ρ=0.7), Seasonal (P=100, α=0.3), Burst (L=10, $p_{b}=0.3$), and Long-memory (H=0.8).

Empirical diagnostics: τ is the smallest lag where normalised autocorrelation (averaged over bits) drops below 1/e; *r* is 1 plus the mean exact-duplicate count in a W=20 forward window.

#### Estimator Cost and Scalability

Both diagnostics are single-pass friendly and cheap relative to any learning workload. The τ estimator requires the bitwise autocorrelation up to a maximum lag *K* (K=500 here): O(nTK) time naively, O(nK) memory with a *K*-length ring buffer per feature, and the lag loop vectorises. The *r* estimator scans a *W*-length forward window per position—O(TW) vector comparisons, or expected O(T) with hashing—using O(Wn) memory. Both are linear in *T*, independent of the alphabet size $N=2^{n}$, and embarrassingly parallel across features; on the longest stream studied (T=22,695) they complete in well under a second on a laptop, so the diagnostic adds negligible overhead for high-volume streaming deployments.

### 5.2. Classical Baselines

Three memory-bounded streaming classifiers cover the key axes:

- **Online SGD**: logistic regression with feature hashing [26,28]; memory O(M).
- **Averaged SGD**: Polyak–Ruppert running average ${\bar{w}}_{t}=\frac{1}{t}\sum_{s}w_{s}$ [27]; lower variance than plain SGD.
- **Count-Min hash classifier**: Count-Min Sketch [23] as a label-averaging lookup table; serves as a collision-limited non-gradient reference (near-chance for M≪N).

Memory budgets range from M=8 to M=256. These three baselines are chosen to span complementary axes of bounded-memory streaming learning—gradient-based (online SGD), variance-reduced gradient-based (averaged SGD), and non-gradient hash-count-based (Count-Min)—while sharing the single-pass, fixed-memory regime that the proxy models. Drift-adaptive methods such as ADWIN-equipped learners or adaptive random forests [30,34] are deliberately excluded from the primary comparison: they respond to non-stationarity by discarding history, which would confound the correlation-budget question the proxy isolates; benchmarking them on the same landscape is a natural extension (Section 9).

### 5.3. Quantum Performance Proxies

Rather than simulating quantum circuits (which would require exponential classical resources at meaningful scale), we compute proxy quantities from the effective-sample model of Section 3.2:

- Oracle MSE proxy: $\epsilon^{2}=C^{2}n\log\left(1/\delta \right)/T_{\mathrm{eff}}$, with C=2, δ=0.05.
- Classification accuracy proxy: $\mathrm{acc}\_\mathrm{proxy}=\max(0.5,\;1-\epsilon^{2})$ (Equation (3)).
- Quantum-memory reference: O(n) qubits (illustrative scaling curve introduced here; Section 3.3).

#### 5.3.1. Justification of Proxy Constants and Forms

Three design choices in the proxy expressions warrant explicit discussion. (i) The constant C=2 in $\epsilon^{2}=C^{2}n\log\left(1/\delta \right)/T_{\mathrm{eff}}$ is a conventional order-unity choice: no specific constant in the cited theory fixes it, and we make no claim that it does. Alternative values C∈{1,1.5,2.5} scale the proxy curve uniformly and preserve all relative orderings between regimes, though the absolute proxy–baseline crossover location shifts with *C* (Table 8). (ii) The confidence parameter δ=0.05 matches the 95% reporting convention used throughout the paper; a 10× change in δ shifts log(1/δ) by a factor of only ≈1.5, which is small relative to the cross-regime differences in $T_{\mathrm{eff}}$ but visible in the absolute crossover location (Table 8). (iii) The seasonal proxy τ=max(1,Pα) is an engineering convenience encoding that periodic streams effectively reset every ∼Pα samples; the long-memory proxy *neglects* the arcsine shrinkage that binarisation induces [53] and is therefore conservative (Section 4). Section 6.9 reports an explicit sensitivity analysis under four alternative τ estimators and three target functions, and shows that the qualitative regime ordering is preserved.

#### 5.3.2. Measured vs. Proxy

**Figure 3:**
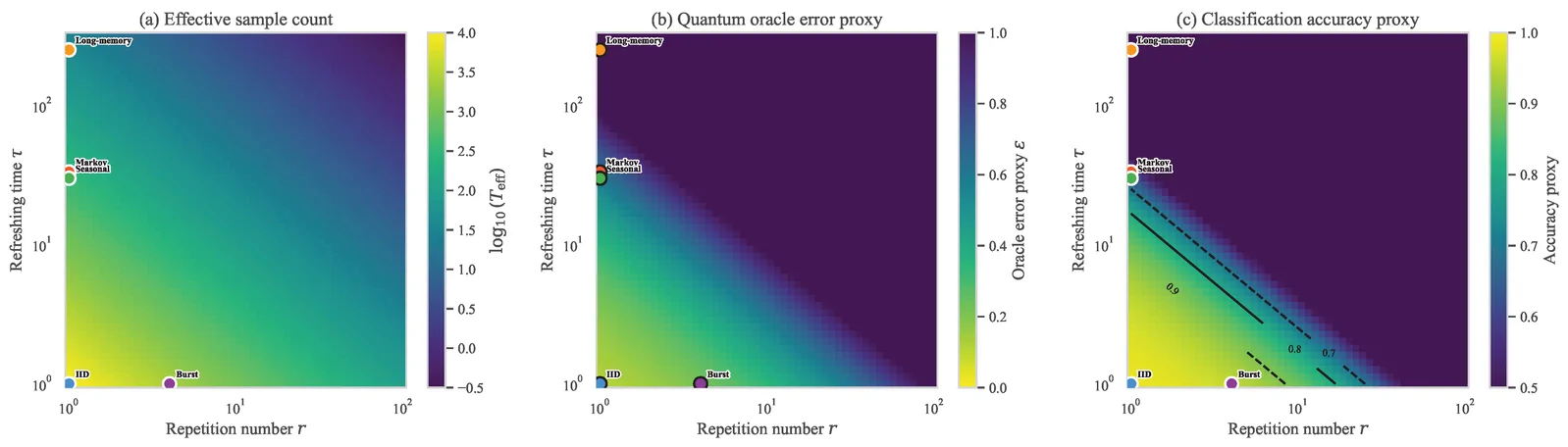
Sensitivity landscape in (τ,r) space (T=10,000, n=10). (**a**) $T_{\mathrm{eff}}=T/\left(\tau r\right)$, log_10_ scale. (**b**) Oracle error proxy ϵ. (**c**) Classification accuracy proxy with contours at 0.7, 0.8, 0.9; colored dots mark each regime’s analytic (τ,r). The landscape is a hypothesis-generation map: contour values are proxy estimates under the modelling assumptions of Section 3.2, not measured quantum performance.

**Empirically measured** (10 seeds, 95% CI): classical accuracy, $\tau_{\mathrm{emp}}$, $r_{\mathrm{emp}}$. **Proxy-estimated** (deterministic): $T_{\mathrm{eff}}=T/\left(\tau r\right)$, $\epsilon^{2}=\min\left(1,\kappa n/T_{\mathrm{eff}}\right)$ with κ≈12, $\mathrm{acc}\_\mathrm{proxy}=\max(0.5,1-\epsilon^{2})$. All figures clearly distinguish the two categories; deterministic landscapes (Figure 3) have no seed dependency.

#### 5.3.3. Reproducibility Parameters

Table 3 consolidates all fixed experimental constants for independent reimplementation.

## 6. Results

We now present nine experiments. Experiments 1–7 are visualisation experiments on the synthetic non-IID regimes, each accompanied by multi-seed classical baselines on the same streams: Experiments 1–2 compare accuracy trajectories; Experiment 3 presents the analytic (τ,r) sensitivity landscape; Experiment 4 contrasts empirical and analytic correlation parameters; Experiment 5 sweeps Markov correlation strength; Experiment 6 examines dimension scaling; and Experiment 7 shows rolling-accuracy dynamics over time. Experiment 8 is a real-stream sanity check on two NAB datasets (Section 6.8) and Experiment 9 is a sensitivity-ablation suite (Section 6.9).

### 6.1. Experiment 1: Classification Accuracy vs. Sample Count

**Figure 1:**
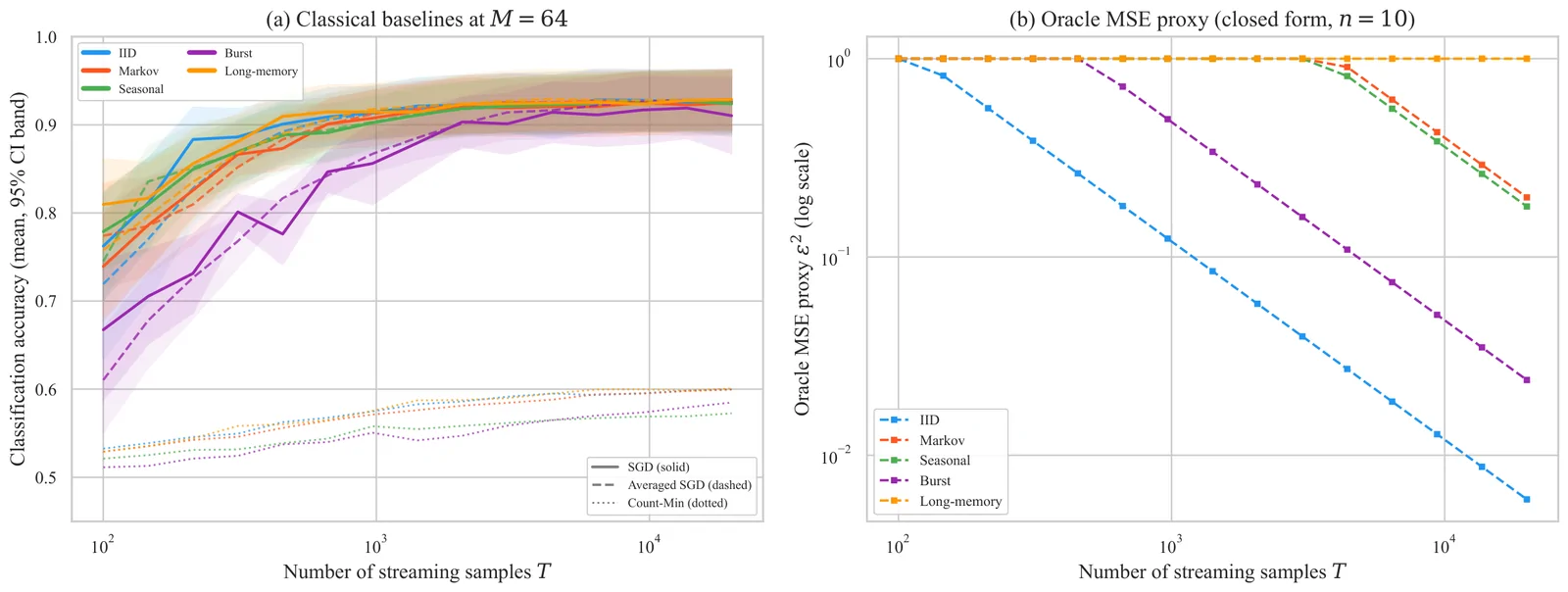
Classification accuracy and proxy error vs. stream length *T*. (**a**) Three classical baselines at M=64 (10-seed mean, ±1.96SE bands): online SGD (solid), averaged SGD (dashed), Count-Min (dotted). (**b**) Oracle MSE proxy $\epsilon^{2}$ (deterministic closed form, n=10), log-log scale; no seed dependence.

Figure 1 shows that all classical baselines converge to 0.88–0.92 accuracy with narrow CI bands. Averaged SGD (Polyak–Ruppert, [27]) produces smoother trajectories, particularly in the burst regime; Count-Min saturates near 0.55–0.60 due to hash collisions at M≪N. The MSE proxy (Panel b) decays as $1/T_{\mathrm{eff}}$: IID and burst show fast decay; for Markov, seasonal, and long-memory, analytic τ inflates $\epsilon^{2}$ enough that the accuracy proxy falls below classical (Section 6.4 quantifies this).

### 6.2. Experiment 2: Classical Accuracy vs. Memory Budget

**Figure 2:**
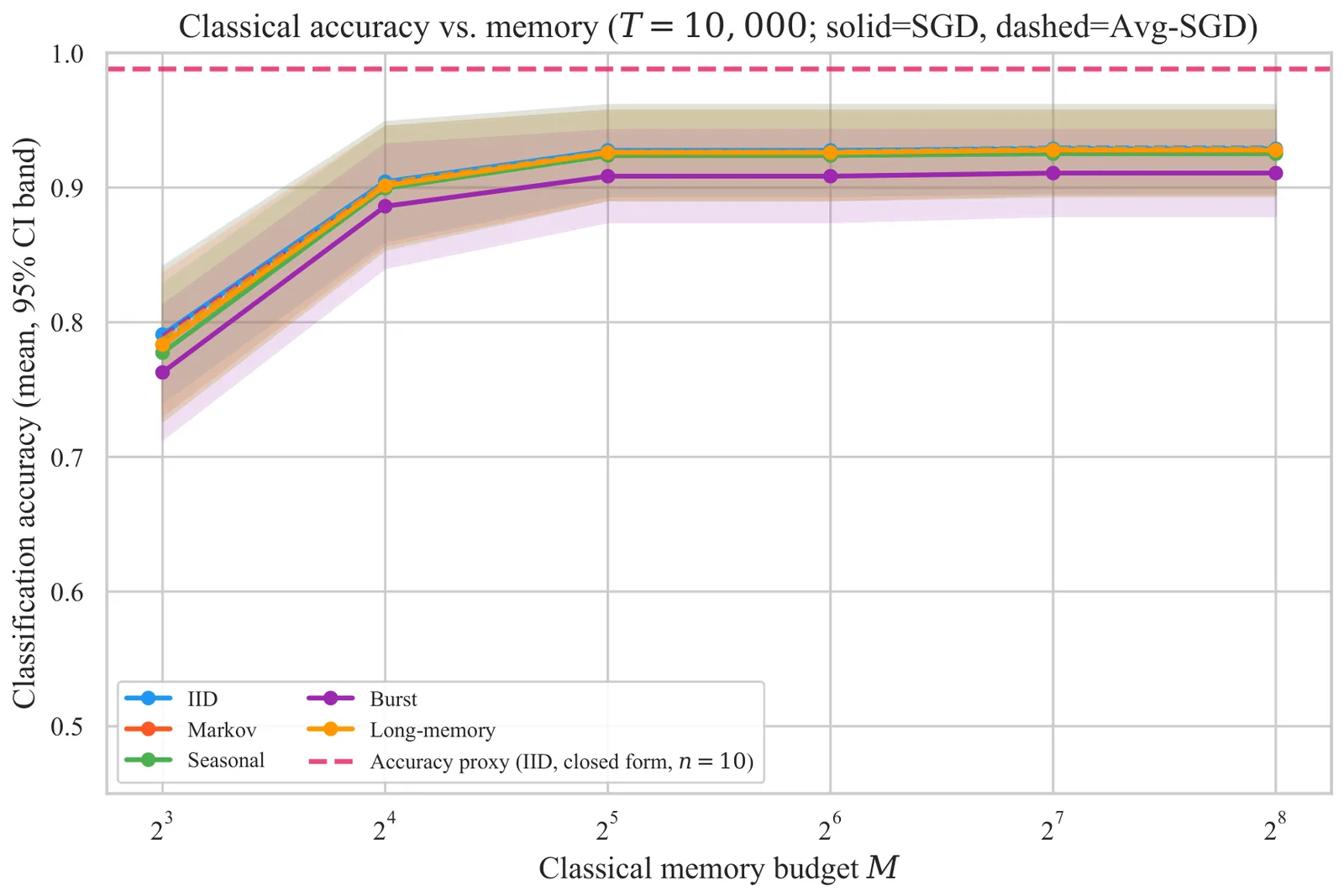
Classical online SGD accuracy vs. memory *M* (T=10,000) compared with the quantum IID proxy (dashed). Classical accuracy saturates at M≥16; under the IID modelling assumptions, the IID proxy lies above the empirical classical curves on this task.

Figure 2 shows that classical accuracy saturates at M≥16, confirming that the SGD learner is sample-limited rather than memory-limited at this scale. Under the IID modelling assumptions, the IID proxy curve (~0.99) lies above the empirical classical curves on this task—a proxy-side ordering, not an implemented algorithmic comparison; the $\Omega (\sqrt{N})$ memory reference is a paper-defined illustrative curve, not a lower bound for this task (Section 3.3).

### 6.3. Experiment 3: Sensitivity Landscape in (τ,r) Space

$T_{\mathrm{eff}}$ decreases diagonally as τ and *r* increase; accuracy contours follow hyperbolas in τ·r space. Under the analytic placements, IID and burst fall in the high-accuracy corner; Markov and seasonal sit between the 0.5 floor and the 0.7 contour (proxy accuracies 0.60 and 0.64, Table 2); long-memory falls in the low-accuracy zone. The empirical placements of the burst and long-memory extremes differ (Section 6.9). Researchers can place estimated (τ,r) values on the landscape to identify comparable synthetic regimes for follow-on empirical or algorithm-level validation.

### 6.4. Experiment 4: Empirical vs. Proxy Correlation Parameters

**Figure 4:**
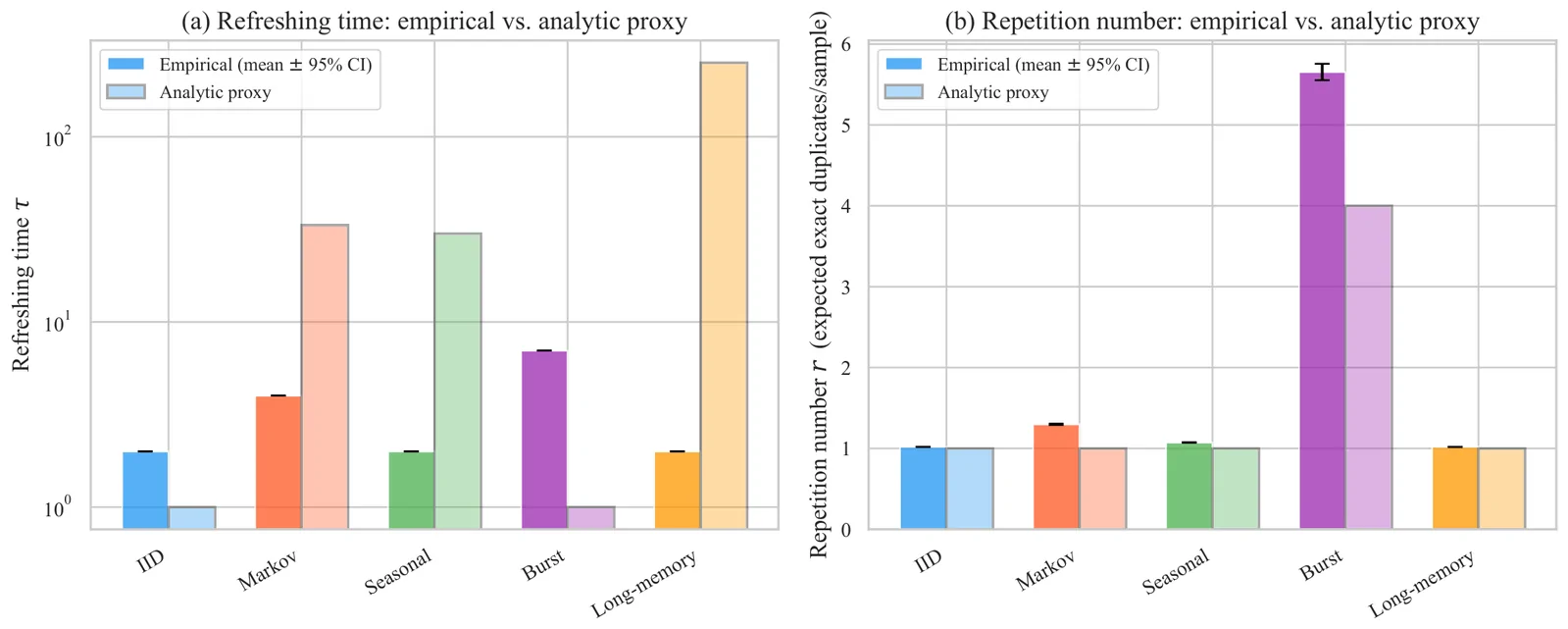
Empirical vs. analytic proxy τ and *r* (10-seed mean ± 95% CI). (**a**) Refreshing time τ: analytic exceeds empirical for Markov, seasonal, long-memory; opposite for IID and burst. (**b**) Repetition number *r*: both quantities in units of expected exact duplicate copies per sample within a W=20 forward window.

Three findings emerge from Figure 4. First, analytic τ systematically overshoots empirical τ: Markov (33 vs. 4, ≈8×), long-memory (251 vs. 2, ≈125×, explained by the arcsine shrinkage from binarisation [53] together with the short-lag character of the estimator [50,51]), and seasonal (30 vs. 2); IID and burst show the opposite sign from finite-sample fluctuations. Second, unit-aligned *r* values agree well: burst empirical r≈5.6 vs. analytic r=4 (factor 1.4×, attributable to window-length choice); all other regimes near 1. Third, IID ($\tau_{\mathrm{emp}}\approx 2$ vs. $\tau_{\mathrm{analytic}}=1$) sets the finite-sample noise floor. These findings confirm that (τ,r) are qualitatively informative but quantitatively loose; family-specific refinements would enlarge the predicted favourable region, and Section 7.3 explains the mechanisms behind the directional gaps.

### 6.5. Experiment 5: Correlation Strength Sweep

**Figure 5:**
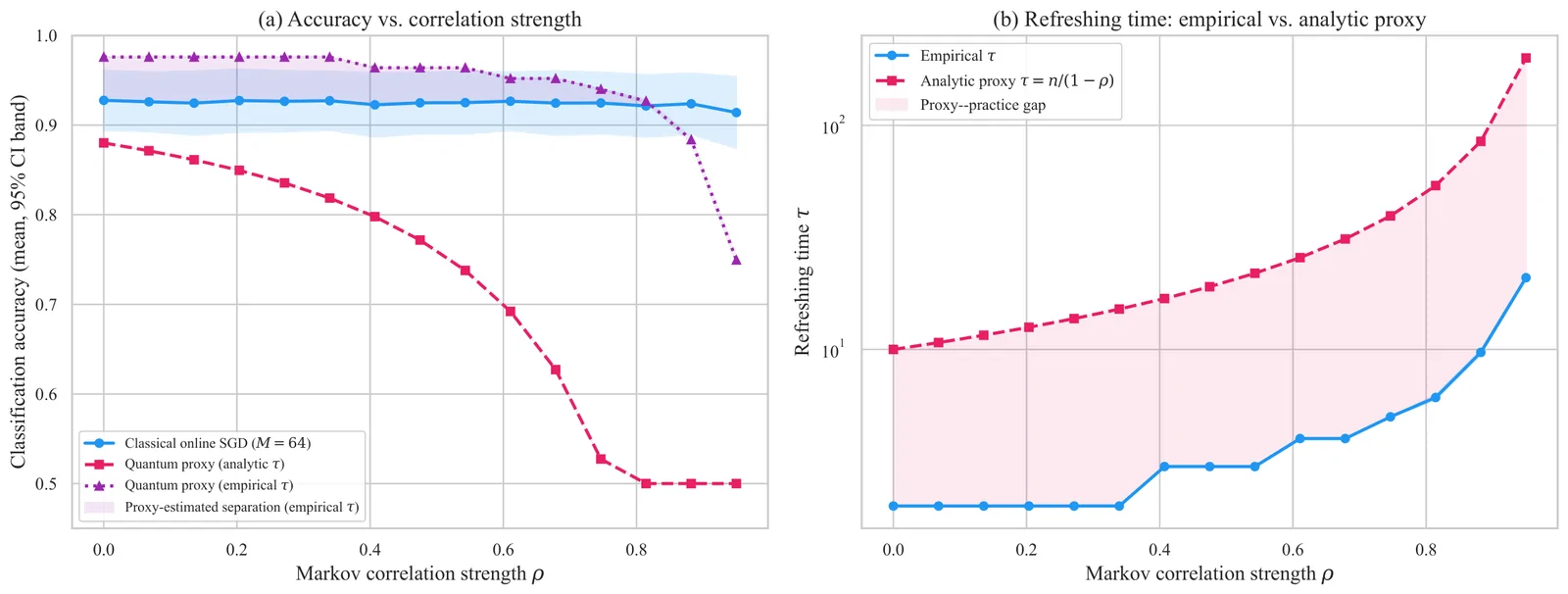
Markov correlation strength sweep (10-seed mean with 95% CI bands). (**a**) Classification accuracy: classical online SGD at M=64 (blue) is approximately flat at ~0.92 across the sweep. The accuracy proxy using the *analytic* τ=n/(1−ρ) (pink, dashed) lies below the classical baseline over the entire sweep and reaches the 0.5 floor by ρ≈0.8. The proxy using the *empirical* τ (purple, dotted) remains above classical until the mean curves cross at $\rho^{*}\approx 0.82$, with the sign reversal established by the interval estimate at ρ≈0.88 (Table 4; the interval is consistent with a reversal as small as 0.003), indicating that tighter family-specific characterisations would enlarge the region in which the proxy model predicts a favourable separation. (**b**) Refreshing time: the proxy–practice gap (shaded) grows with ρ, showing that the analytic proxy is increasingly conservative under stronger correlation. Marginal CI bands of the classical and empirical-τ proxy curves overlap; the sign of their difference is resolved by the paired analysis of Table 4, not by band separation.

Figure 5 examines how the classical-vs-proxy separation evolves as the Markov correlation strength ρ is varied continuously from 0 (IID) to 0.95 (strong correlation), comparing the classical SGD baseline against the quantum proxy using both the *analytic* τ and the *empirically measured* τ.

**Table 4:** Classical-vs-proxy comparison across representative Markov correlation strengths ρ: empirical classical online-SGD accuracy at M=64 and the heuristic quantum accuracy proxy using the empirically measured τ (10-seed mean ± 95% CI half-width). Tests are computed at five pre-specified targets ρ∈{0,0.3,0.5,0.7,0.9}, snapped to the nearest of the 15 sweep grid points; the Holm family is these five. $\hat{\Delta }$ is the Hodges–Lehmann (pseudomedian) estimate of the paired (proxy−classical) difference with its exact signed-rank interval (achieved coverage 95.1% at n=10); $r_{\mathrm{rb}}$ is the matched-pairs rank-biserial correlation; *p* is the two-sided paired Wilcoxon signed-rank *p*-value, reported descriptively. Marginal ± values are 1.96×SE (normal approximation); a proxy half-width of 0.000 is exact—all ten seeds return the same integer τ, reflecting lattice quantisation of a discrete estimator, not proxy precision. A positive $\hat{\Delta }$ means only that the proxy estimate exceeds the empirical classical baseline, not realised quantum algorithmic superiority. Under a Holm correction across the five points, no comparison falls below the 0.05 family-wise level (smallest adjusted p=0.098); the evidence for the crossover is the monotone sign reversal of $\hat{\Delta }$ and its interval estimates, not any single test.

ρ	Classical SGD	Proxy (Emp. τ)	$\hat{\Delta }$ [95% CI]	$r_{\mathrm{rb}}$	***p***
0.00	0.928±0.034	0.976±0.000	+0.042[+0.011,+0.087]	+0.82	0.020
0.27	0.927±0.034	0.976±0.000	+0.042[+0.012,+0.090]	+0.78	0.027
0.48	0.925±0.034	0.964±0.000	+0.031[+0.002,+0.080]	+0.71	0.049
0.68	0.925±0.036	0.952±0.000	+0.019[−0.011,+0.069]	+0.35	0.375
0.88	0.924±0.034	0.884±0.004	−0.046[−0.079,−0.003]	−0.75	0.037

Panel (a) reveals a key contrast between the two τ conventions. The accuracy proxy computed with the *analytic* τ=n/(1−ρ) lies below the measured classical baseline over the entire sweep (proxy 0.88 at ρ=0 against classical 0.928) and reaches the 0.5 floor by ρ≈0.8. Using *empirical* τ instead, the proxy remains above 0.9 for ρ≤0.8 and above the classical baseline across most of the sweep. Under the empirical-proxy model, the paired proxy − classical difference declines from +0.042 at ρ=0 (Hodges–Lehmann estimate; exact 95% CI [+0.011,+0.087]) to +0.019 at ρ=0.68 (CI [−0.011,+0.069], spanning zero) and reverses sign to −0.046 by ρ=0.88 (CI [−0.079,−0.003]). The mean proxy and classical curves cross between the ρ=0.81 and ρ=0.88 grid points (interpolated $\rho^{*}\approx 0.82$ under the operating constants; the pivotal grid-point difference is within seed noise, so $\rho^{*}$ should be read as a bracket rather than a point, and Section 6.9 quantifies how it moves with *C* and δ). The hypothesised proxy-favourable region is closed by ρ≈0.88, where even the empirical autocorrelation becomes severe. Table 4 reports the full interval estimates and effect sizes.

Panel (b) quantifies the source of this discrepancy: the analytic proxy τ=n/(1−ρ) grows as a pole at ρ=1, while the empirical τ grows much more gradually. The shaded proxy–practice gap widens by over an order of magnitude at ρ=0.9. This gap is the primary driver of the pessimistic quantum performance proxies observed in Experiments 1–3, and its characterization is one of the central contributions of this paper.

#### What These Tests Do and Do Not Establish

These comparisons pair an empirical, stochastic classical learner with a model prediction, not with a second implemented algorithm: for each ρ, the paired observations are the per-seed classical accuracy and the same seed’s proxy value, which is a closed-form function of that seed’s empirically measured τ. The proxy’s seed-to-seed variability is therefore inherited entirely from the τ estimator (negligible at ρ≤0.68, ±0.004 at ρ=0.88), not from any stochastic training process. The tests consequently quantify whether the sign is of the classical—proxy offset is stable across seeds—they do not, and cannot, establish that one algorithm outperforms another, and no quantum algorithm is implemented anywhere in this comparison. We accordingly interpret Table 4 through the interval estimates, with two caveats stated up front. First, because the proxy arm is near-constant, $r_{\mathrm{rb}}$ is scale-free and indexes *sign concordance across seeds* rather than magnitude: $r_{\mathrm{rb}}\geq +0.71$ means at most two of ten seeds are discordant, and all magnitude information lives in $\hat{\Delta }$ (about four accuracy points at mild correlation). Second, the τ estimator is integer-valued and one lattice step changes the proxy by exactly 0.012: shifting $\hat{\tau }$ by +1 at ρ=0.48 moves that row’s interval from [+0.002,+0.080] to [−0.010,+0.068], across zero, so the zero-exclusion at ρ=0.48 is not robust to a one-lag change of estimator (the ρ≤0.27 and ρ=0.88 intervals are). With those caveats, the pattern is sign-concordant positive differences at mild correlation, indistinguishable from zero at ρ=0.68, and sign-concordant negative at ρ=0.88 ($r_{\mathrm{rb}}=-0.75$, CI excluding zero). The unadjusted *p*-values are reported for completeness; under a Holm correction across the five pre-specified points none falls below the 0.05 family-wise threshold at n=10 seeds—an empirical outcome of one-to-two discordant seeds, not a design ceiling, since the smallest attainable adjusted *p* at n=10 is 0.0098—so we treat the sign reversal and its interval estimates, not statistical significance, as the finding. The crossover location itself is the deterministic solution of $\mathrm{acc}\_\mathrm{proxy}\left(\hat{\tau }\left(\rho \right)\right)={\bar{a}}_{\mathrm{classical}}$; its uncertainty is inherited from $\hat{\tau }$ and from the constants (C,δ), not from the tests.

### 6.6. Experiment 6: Dimension Scaling

**Figure 6:**
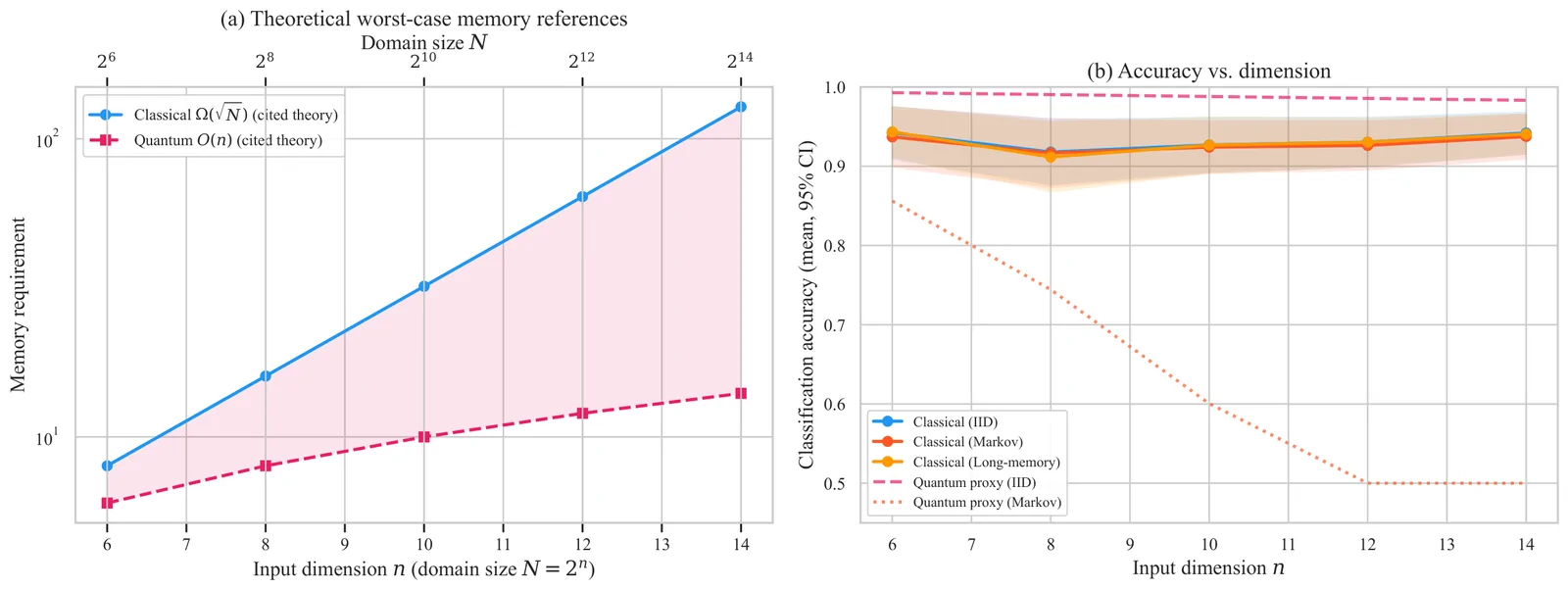
Dimension scaling analysis. (**a**) Illustrative memory-scaling reference (paper-defined; no measured content): a classical curve $\Omega \left(\sqrt{N}\right)=\Omega \left(2^{n/2}\right)$ versus a quantum curve O(n). Their exponential divergence is a visual scaling contrast, not an established separation or a witnessed empirical floor for any baseline studied here (Section 3.3). To preserve the submitted artwork, the panel’s legacy internal “cited theory” labels remain; in this version they indicate only general literature motivation and do not attribute these chosen curves to a specific theorem. (**b**) Classification accuracy vs. dimension *n*: classical online-SGD accuracy for IID, Markov, and long-memory data (solid lines; 10-seed mean, ±1.96SE bands) compared with deterministic accuracy proxies for IID and Markov (dashed). The IID proxy is consistently near 1.0 (a task-level generalisation trend, not evidence of algorithmic feasibility; Section 3.3); the analytic-τ Markov proxy degrades with dimension (because $\tau_{\mathrm{Markov}}=n/\left(1-\rho \right)$ grows with *n*, reducing $T_{\mathrm{eff}}$), while classical accuracy is approximately stable across the range studied.

Figure 6 illustrates the exponential divergence of the paper-defined quantum-vs-classical memory reference curves with data dimension. No memory lower bound is measured empirically in this experiment: the curves in panel (a) are illustrative quantities, and no result here constitutes an empirical memory floor for online SGD, averaged SGD, or Count-Min.

Panel (a) shows that at n=14 (N=16,384) the illustrative classical memory curve exceeds 128 while the illustrative quantum curve is 14 qubits; their exponential divergence in *n* is a paper-defined visual reference, not a cited lower bound or an empirical floor measured for any baseline here (Section 3.3). Panel (b) shows that classical SGD accuracy is approximately stable across n∈[6,14], while the quantum IID proxy is consistently near 1.0. This flatness is a property of the task-level $\sqrt{n/T_{\mathrm{eff}}}$ rate and should not be read as algorithmic feasibility at higher dimensions (Section 3.3). The quantum Markov proxy *decreases* with dimension because $\tau_{\mathrm{Markov}}=n/\left(1-\rho \right)$ grows linearly with *n*; family-specific proxy refinements are therefore most useful at higher dimensions.

### 6.7. Experiment 7: Rolling Accuracy Dynamics

**Figure 7:**
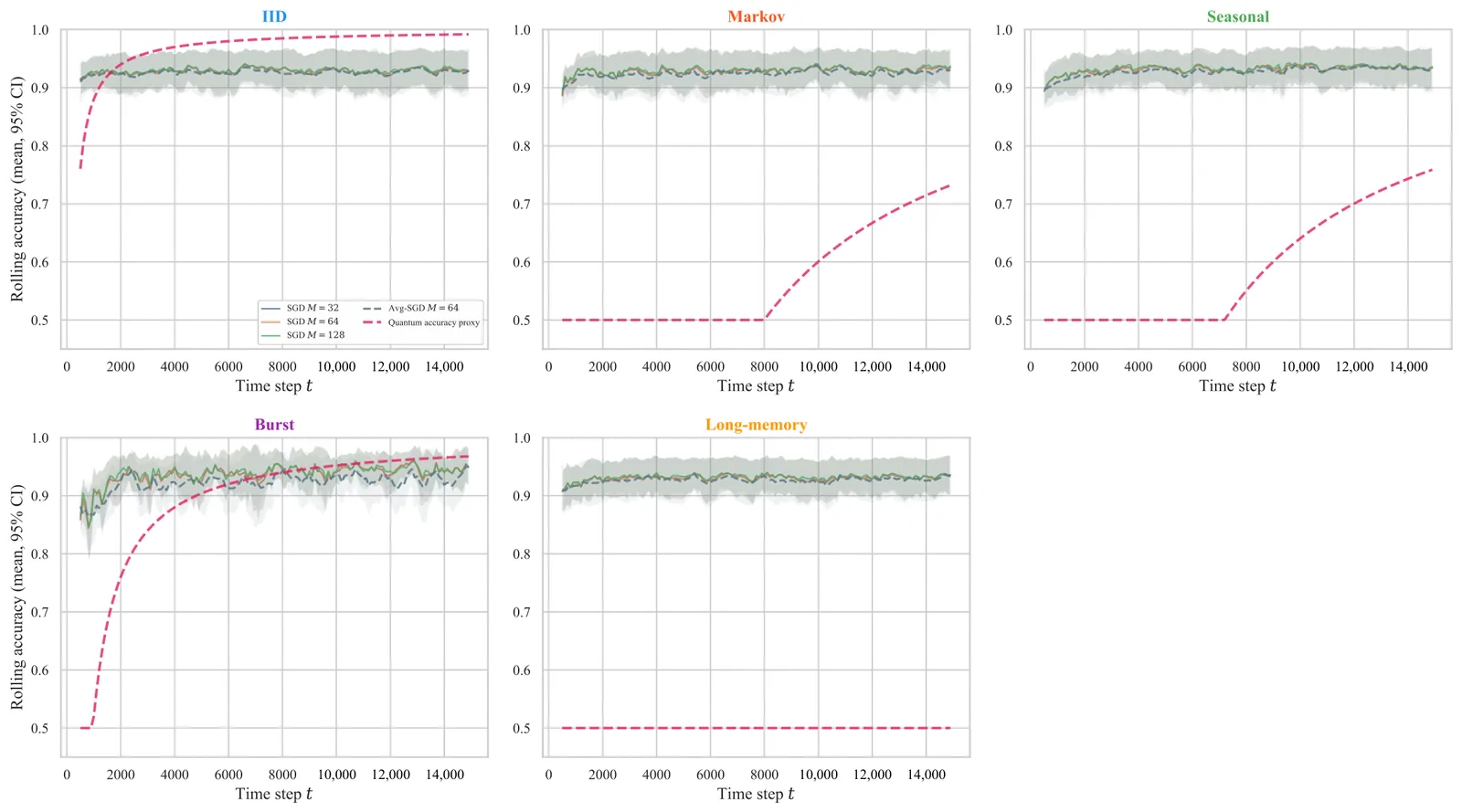
Rolling accuracy (window =500) per regime; 10-seed mean ± 95% CI. Classical SGD at M∈{32,64,128} (solid), averaged SGD M=64 (dashed grey), accuracy proxy (dashed pink; analytic τ). Under IID and burst the proxy reaches or exceeds classical by horizon end; under Markov and seasonal it rises more slowly and remains below classical (conservative analytic τ); under long-memory the analytic-τ proxy is pinned at 0.5 throughout (the empirical-τ placement of this regime differs; Section 6.9).

Figure 7 confirms the regime-dependent pattern of Experiments 1 and 5: IID and burst show higher proxy-estimated trajectories; Markov and seasonal show a visible gap attributable to the conservative analytic τ (a tighter family-specific proxy would shift the trajectory upward, as the Markov sweep confirms); long memory exhibits the 0.5 saturation floor and persistent low-frequency classical oscillations characteristic of long-range dependence.

### 6.8. Experiment 8: Real-Stream Sanity Check

The synthetic experiments above leave open whether the empirical τ and *r* estimators apply usefully to real streams. To answer this we apply the proxy framework end-to-end to two publicly available real streams from the Numenta Anomaly Benchmark [54] (NAB; MIT licence) and report what is observed. No claim of quantum advantage is made; the contribution of this experiment is the successful application of the diagnostic workflow outside the synthetic regime, not a comparison against a realised quantum algorithm. The two streams were selected on structural grounds (contrasting correlation regimes: transport-traffic seasonality versus industrial-sensor drift) before any baseline was run, and all outcomes that emerged are reported; no registration artifact exists for this choice, so it is a disclosure, not a pre-registration claim.

#### 6.8.1. Datasets

(i) *NYC Taxi*: T=10,320 half-hour pickup counts in New York City (1 July 2014 to 31 January 2015). Combines strong daily/weekly seasonality with documented burst events (NYC Marathon, Thanksgiving, and snowstorms); overlaps the synthetic seasonal + burst + low-order Markov regimes. (ii) *Machine Temperature*: T=22,695 five-minute readings from an industrial machine sensor preceding a documented system failure. Exhibits slow drift over hours to days, plus localised spike events; overlaps the long-memory + burst regimes.

#### 6.8.2. Binarisation Choice

Each continuous series is encoded into n=6 binary features (matched to Section 6.9): a 3-bit time-of-day bucket, a weekend flag, and two trend bits (current and lag-1 indicator of whether the value exceeds a 7-day rolling median; the rolling window is 336 bins for NYC Taxi at 30-min resolution and 2016 bins for Machine Temperature at 5-min resolution). The target $y_{t}$ is a simple rare-event classification proxy: $y_{t}=1$ iff the next-bin value is at or above the 80th percentile of the full series. *This is a per-stream high-activity threshold and not the original NAB anomaly label.* The 80th percentile is chosen because it produces a non-trivial ∼20%/80% class imbalance that exposes both majority-baseline behaviour and the rare-event regime; this target is intentionally imbalanced and not a balanced classification task. To prevent accuracy from masking majority-class memorisation, we report balanced accuracy and $F_{1}$ of the positive (rare) class alongside accuracy in Table 6. The recorded threshold sensitivity spans positive rates 0.30/0.20/0.10 at q∈{0.70,0.80,0.90} (threshold values in the released JSON); baseline metrics are reported at q=0.80 only. One further design simplification is disclosed here: the threshold is computed from the *full* series, so the target definition uses information not available prequentially at each step. The feature stream and the (τ,r) estimates involve no such look-ahead, and the landscape placement is target-free, so this affects only the classical-baseline reference numbers; a threshold calibrated on an initial interval, or updated from previously observed samples only, is the rigorous prequential design and is adopted as future work. Binarisation discards information, so the resulting empirical τ and *r* should be read as lower bounds on the correlation lengths in the underlying continuous process.

#### 6.8.3. Procedure

Empirical τ and *r* are computed using the same estimators as the synthetic experiments (Section 5), on the *temporally ordered* stream. The three classical baselines are run with M=64 on seed-permuted prequential orders (10 random shuffles of the fixed series; ±1.96SE intervals). Two consequences of this design are stated plainly: the intervals measure ordering variability of a single fixed dataset—not sampling uncertainty about the stream, sensor, or domain—and, because permutation removes the temporal dependence that (τ,r) quantify, the classical accuracies are upper-optimistic relative to the ordered stream. The proxy accuracy is $\mathrm{acc}\_\mathrm{proxy}=\max(0.5,1-C^{2}n\log\left(1/\delta \right)/T_{\mathrm{eff}})$ with C=2, δ=0.05.

#### 6.8.4. Findings (Figure 8, Table 5 and Table 6)

The two streams land on opposite sides of the proxy-favourable region in (τ,r) space, and the imbalance-aware metrics expose effects that accuracy alone hides:

- **NYC Taxi** (proxy-favourable region): $\tau_{\mathrm{emp}}=6.0$, $r_{\mathrm{emp}}=2.77$, $T_{\mathrm{eff}}=621$, proxy =0.884. Count-Min reaches accuracy 0.865±0.016, balanced accuracy 0.777±0.042, and $F_{1}=0.644\pm 0.062$, materially above majority on every metric (though with wide ordering-to-ordering spread: per-ordering $F_{1}$ ranges 0.43–0.72). Both SGD variants fall *below* the 0.800 majority baseline on accuracy (0.670±0.006 and 0.673±0.004), and Averaged-SGD’s balanced accuracy (0.498±0.012, interval straddling 0.50) is indistinguishable from chance—under this binarisation the hashed-linear learners do not recover the rare class.
- **Machine Temperature** (proxy-unfavourable region): $\tau_{\mathrm{emp}}=32.0$, $r_{\mathrm{emp}}=13.55$, $T_{\mathrm{eff}}=52$, proxy =0.500 (saturated at the chance floor). All three classical baselines have accuracy at or slightly above/below the majority value (0.724, 0.735, 0.801 against 0.800), balanced accuracy of 0.55, 0.54, and 0.56, and $F_{1}\in \left[0.23,0.27\right]$. The accuracy levels are therefore produced largely by majority-class prediction; the balanced accuracies exceed the 0.50 chance level by a statistically resolvable but practically negligible margin.

**Table 5:** Real-stream sanity check, landscape coordinates: empirical (τ,r), proxy accuracy, and classical-baseline accuracy (10-ordering mean) for two NAB datasets; the majority baselines appear in Table 6. The proxy places NYC Taxi in a proxy-favourable region and Machine Temperature in a proxy-unfavourable region (proxy saturates at 0.5). Imbalance-aware metrics for the same runs are reported in Table 6.

Dataset	*T*	τ	*r*	$T_{\mathrm{eff}}$	Proxy	SGD	Avg-SGD	Count-Min
NYC Taxi	10,320	6.0	2.77	621	0.884	0.670	0.673	0.865
Machine Temp.	22,695	32.0	13.55	52	0.500	0.724	0.735	0.801

**Table 6:** Imbalance-aware metrics for the real-stream sanity check (10-ordering mean ±1.96SE). Accuracy alone is misleading at a ∼20%/80% positive/negative split, so balanced accuracy and $F_{1}$ of the positive (rare) class are reported alongside it. Majority-baseline rows are determined by the 80th-percentile target construction (base rate ≈0.20) and are identical for both streams by design; balanced accuracy 0.500 and $F_{1}=0.000$ are the analytic values for a constant classifier. The proxy is an accuracy projection only; balanced accuracy and $F_{1}$ are not targets the proxy attempts to match.

Dataset	Method	Accuracy	Balanced Acc.	$F_{1}$ (Positive)
NYC Taxi	Majority	0.800	0.500	0.000
	Online-SGD	0.670±0.006	0.535±0.004	0.273±0.006
	Averaged-SGD	0.673±0.004	0.498±0.012	0.199±0.025
	Count-Min	0.865±0.016	0.777±0.042	0.644±0.062
Machine Temperature	Majority	0.800	0.500	0.000
	Online-SGD	0.724±0.001	0.550±0.019	0.269±0.047
	Averaged-SGD	0.735±0.002	0.538±0.016	0.234±0.049
	Count-Min	0.801±0.001	0.560±0.021	0.228±0.075

**Figure 8:**
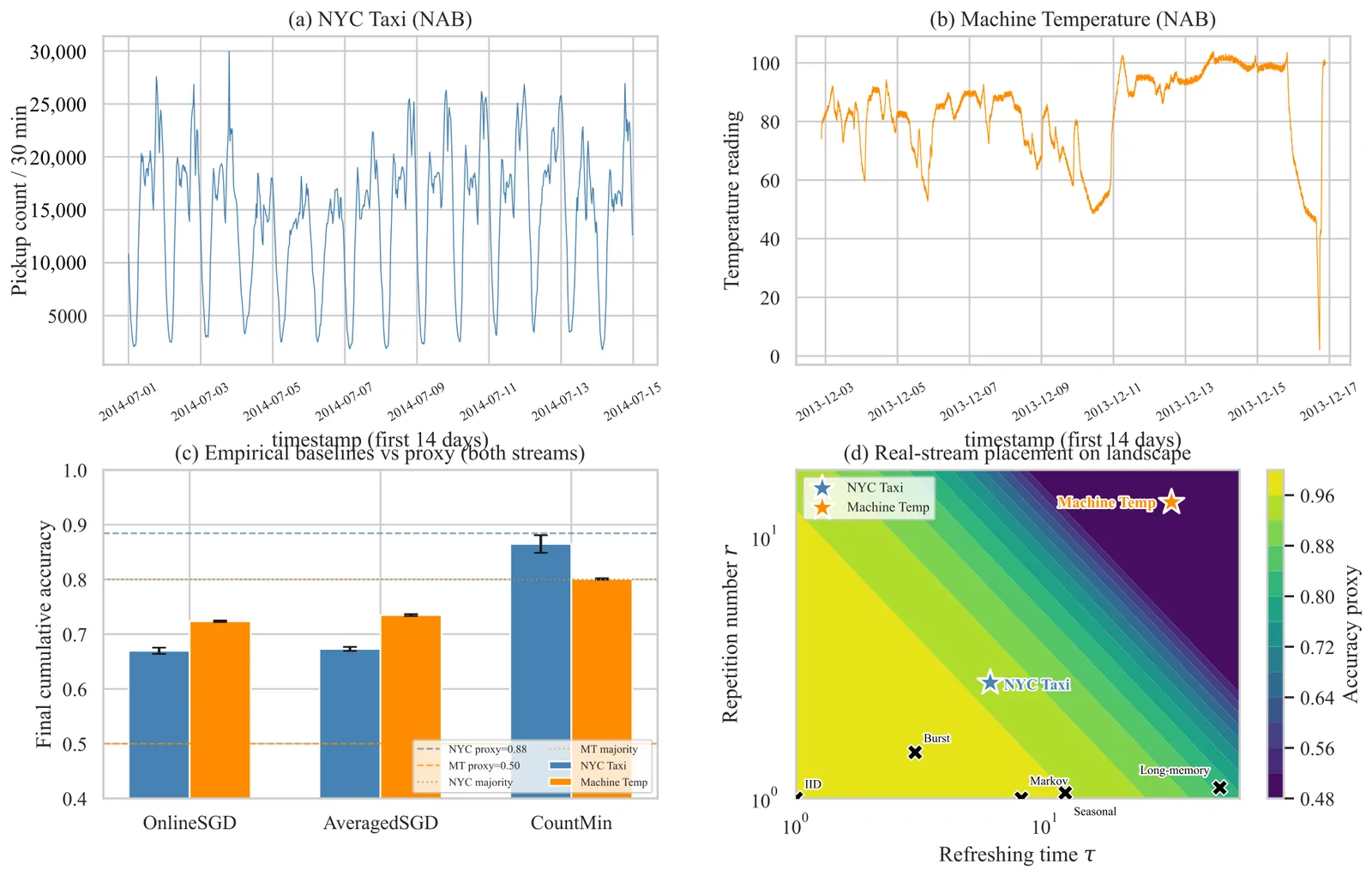
Real-stream sanity check (two NAB datasets). (**a**,**b**) Raw series for the first 14 days of each dataset. (**c**) Final cumulative classical baseline accuracy (10-seed mean ± 95% CI) for both streams; dashed lines are the deterministic proxy values, dotted lines are the majority baseline. (**d**) Both real-stream points (coloured stars, plotted at their measured coordinates) on the (τ,r) landscape, alongside *indicative* synthetic-regime positions (black crosses; schematic placements for orientation, not the measured coordinates of Table 2 or Figure 4). NYC Taxi sits at (τ,r)=(6.0,2.77), $T_{\mathrm{eff}}=621$, in a moderately proxy-favourable region; Machine Temperature sits at (32.0,13.55), $T_{\mathrm{eff}}=52$, in the proxy-unfavourable upper-right region where the proxy saturates. The stars are computed at n=6 and the underlying contours at n=10; because contour values depend on *n* and *T*, the overlay supports qualitative placement only. Imbalance-aware metrics for the same runs are reported in Table 6.

#### 6.8.5. Why Count-Min Looks Strong on NYC Taxi

The near-proxy accuracy of Count-Min on NYC Taxi has a structural explanation that is worth stating up front, because otherwise the apparent agreement could be misread as evidence that the proxy is “correct”. With n=6 binary features only $2^{n}=64$ feature vectors exist, and many of them repeat in the data; Count-Min can therefore essentially memorise per-bucket positive rates and predict the majority class for each bucket, which is enough to reach $F_{1}=0.644$ at this binarisation granularity. SGD with hashed-linear features cannot represent the same per-bucket conditional distribution as efficiently. The Count-Min result should be interpreted as a memorisation-friendly bounded-memory baseline under coarse binarisation, not as general evidence that the proxy predicts all classical learners. As *n* grows, $2^{n}$ outpaces practical Count-Min table sizes and this memorisation route disappears.

#### 6.8.6. Interpretation

Three observations. (i) The empirical τ and *r* estimators apply unchanged to both real streams and place each stream in the regime that matches its underlying structure: NYC Taxi has moderate τ (multiple-bin daily/weekly correlation) and moderate *r* (modest exact-feature-vector repetition at n=6); Machine Temperature has high τ (slow multi-hour drift) and very high *r* (5-min sensor data has many near-identical consecutive feature vectors). (ii) On NYC Taxi, Count-Min materially exceeds the majority baseline on balanced accuracy (0.777 vs. 0.500) and on $F_{1}$ (0.644 vs. 0.000), so the accuracy match between Count-Min and the proxy reflects genuine rare-class learning rather than majority memorisation alone, even though the underlying mechanism is the bounded-*n* memorisation route described above. (iii) On Machine Temperature, the saturated proxy is exceeded by all classical baselines on accuracy, but the imbalance-aware metrics show that the accuracy levels are produced mostly by majority-class prediction: balanced accuracy is at most 0.56 and $F_{1}$ is below 0.30 for every classical baseline. Read strictly as an accuracy projection, the proxy is *falsified* on this stream (0.500 predicted against 0.72–0.80 observed); the diagnostic reading—that at $T_{\mathrm{eff}}=52$ no sample-limited estimator should be expected to recover the *rare class*—reinterprets the saturated value as a rare-class-learnability indicator, which is a change of metric and is offered as interpretation, not confirmation. For symmetry, the proxy’s miss on NYC Taxi is 0.019 against accuracy and 0.107 against balanced accuracy. We also state plainly that the favourable-side separation between the two streams rests on Count-Min alone; the hashed-linear learners do not corroborate it, consistent with the scope boundary that the proxy does not predict per-learner outcomes (Section 7.4).

#### 6.8.7. Encoding and Resolution Sensitivity

Because four of the six binary features are deterministic calendar fields, the measured (τ,r) could in principle reflect the ratio of sampling rate to encoder field width rather than the underlying signal. To check this, we re-encoded the same raw series across sampling resolutions and time-of-day bucket widths and recomputed $\left(\tau ,r,T_{\mathrm{eff}}\right)$ and the proxy with the unchanged estimators (deterministic re-analysis; no training). Table 7 shows that absolute values do move with the encoder—as they must, since the diagnostic characterises the stream *as encoded and fed to the learner*—but the ordering never reverses: NYC Taxi’s proxy value exceeds Machine Temperature’s in every one of the ten configurations, including at matched 30-min resolution (0.884 vs. 0.576) and matched 60-min resolution (0.908 vs. 0.719). Machine Temperature leaves proxy saturation only when downsampled 6–12×, which discards precisely the fast-timescale redundancy that the diagnostic is designed to count. Placements should accordingly be read as encoder-conditional but ordering-stable.

The two streams together demonstrate the (τ,r) *placement* workflow: the estimators apply unchanged to both streams and place them at opposite ends of the $T_{\mathrm{eff}}$ range (621 vs. 52), an ordering that is stable across encoder configurations (Table 7). We do not claim the placement predicts classical performance—on balanced accuracy, both SGD variants score marginally *higher* on the nominally unfavourable stream, and only Count-Min (via the memorisation route above) separates the two—and the imbalance-aware metrics are consistent with the placement reading rather than proof of it. With only two datasets, however, this experiment is illustrative rather than statistically representative: it demonstrates that the diagnostic workflow runs end-to-end on real telemetry and produces interpretable placements, but it cannot establish that those placements generalise across stream domains. Broader real-stream coverage across financial, network traffic, cybersecurity, and additional sensor domains remains future work (Section 9).

### 6.9. Experiment 9: Proxy-Sensitivity and Estimator Ablations

To address the concern that several proxy choices appear ad hoc, we vary five ingredients of the pipeline and report whether the qualitative ordering of regimes on the (τ,r) landscape is preserved. The first four ablations use T=4096, W=20, and a feature dimension n=6 chosen to match the real-stream case study (Section 6.8) for direct comparability; the fifth (the *C*–δ analysis) re-evaluates the Markov sweep of Section 6.5 at its native $T=10^{4}$, n=10; the synthetic main experiments use n=10, so absolute proxy values in this section are not comparable across sections. What this section establishes is that the regime ordering induced by the *empirical-τ estimator family* is stable across the four estimators within it. That ordering differs from the analytic-τ ordering of Table 2 at the two extremes—long-memory moves from worst (analytic) to the best tier (empirical, the short-lag artifact of Section 7.3), while burst moves from second-best to saturated worst (its measured τ·r is the largest of any regime)—and this estimator dependence is itself a finding, not a defect to be hidden.

#### 6.9.1. Alternative τ Estimators

Four definitions are compared: the default (1/e ACF crossing), an aggressive variant (1/2 ACF crossing), the integrated absolute ACF up to first non-positive lag, and an AR(1) effective lag $-1/\log|\rho_{1}|$. The qualitative ordering is preserved across all five regimes: Burst always has the largest τ (3.3–7.0), IID and long-memory always the smallest (1.0–2.0), Markov consistently moderate (2.8–4.0). Resulting proxy accuracies (Table 9) shift by at most 0.04 between estimators for the four non-saturated regimes; the Burst regime, which sits at the 0.5 saturation floor at T=4096, moves by 0.155 under the AR(1) estimator because that estimator captures only the lag-1 dependence.

#### 6.9.2. Forward-Window Size *W*

Repeating the *r* estimator at W∈{10,20,50} yields a monotone increase in *r* (more chances to find duplicates) and a corresponding monotone decrease in $T_{\mathrm{eff}}$. The relative ordering of regimes by *r* is insensitive to *W* over the range tested; this establishes ordering stability, not that W=20 is uniquely representative.

#### 6.9.3. Stream Length *T*

Varying T∈{1024,2048,4096,8192} yields the expected scaling in $T_{\mathrm{eff}}$. For Burst, the proxy emerges from saturation only at T≥8192, indicating sample-size sensitivity for highly repetitive regimes.

#### 6.9.4. Target Function

This ablation uses a colder protocol than the main experiments—prequential (test-then-train) accuracy over the full stream at learning rate 0.01 and T=4096, versus held-out evaluation at learning rate 0.05 and $T=10^{4}$ in Section 6, Section 6.1, Section 6.2, Section 6.3, Section 6.4, Section 6.5—so absolute SGD levels here are not comparable to Table 4. Under this protocol, on the linear target the SGD baseline degrades to near-chance (≈0.5) for these synthetic streams and the small n=6 feature set; on a threshold target (y=1 iff $\sum_{i}x_{i}\geq n/2$) SGD attains ≈0.65 across all regimes; on the parity target SGD remains near chance. The proxy model is target-independent (it depends only on $T_{\mathrm{eff}}$), so this ablation isolates the gap between the proxy framework and the empirical learner’s task-specific capability. This is a scope boundary, stated plainly: classical learner performance is strongly target-dependent, while the proxy depends only on (τ,r,T) and is target-independent by construction. The proxy therefore cannot universally predict downstream classifier performance; it characterises the correlation structure of the stream—the effective sample budget available to *any* bounded-memory learner—not task-specific learnability. Predictions read off the landscape are statements about correlation-driven budget erosion and must not be interpreted as accuracy forecasts for a particular classifier–target pair (Section 7.4).

#### 6.9.5. Proxy Constants *C* and δ

Because the proxy is a closed-form function of the measured (τ,r), its sensitivity to the constants requires no new stochastic runs: we re-evaluate the accuracy proxy on the same per-seed empirical τ values from the Markov sweep (Section 6.5) over the grid C∈{1,1.5,2,2.5}, δ∈{0.01,0.05,0.1}, and record where the mean proxy curve crosses the mean classical curve. Table 8 shows that the regime *ordering* is invariant—as it must be, since *C* and δ enter only through the multiplicative factor $\kappa =C^{2}\log\left(1/\delta \right)$, a monotone rescaling—but the *absolute* crossover location $\rho^{*}$ moves substantially: from $\rho^{*}\approx 0.38$ at the most pessimistic setting (C=2.5,δ=0.01) to beyond the sweep range at C=1. At the paper’s operating point (C=2,δ=0.05), $\rho^{*}\approx 0.82$. The landscape should therefore be read *ordinally*: relative placements of regimes and streams are robust to the constants, while any absolute boundary—including the $\rho^{*}$ values above—is conditional on the chosen (C,δ).

**Table 8:** Sensitivity of the proxy–baseline crossover $\rho^{*}$ (Markov sweep, empirical τ, mean curves) to the proxy constants *C* and δ. Entries are interpolated on the 15-point sweep grid; “>0.95” means the mean proxy curve remains above the mean classical curve over the entire sweep. Regime ordering is invariant across the grid; the absolute crossover is not. The paper’s operating point (C=2, δ=0.05) is set in bold.

	δ=0.01	δ=0.05	δ=0.10
C=1.0	0.93	>0.95	>0.95
C=1.5	0.84	0.89	0.92
C=2.0	0.61	0.82	0.86
C=2.5	0.38	0.61	0.76

**Table 9:** Proxy-sensitivity ablation: alternative τ estimators on synthetic streams (T=4096, W=20, n=6). Qualitative ordering of regimes by τ is preserved across all four estimators; proxy accuracy changes by at most 0.04 for the non-saturated regimes, while Burst (at the 0.5 floor) moves 0.155 under the AR(1) estimator. Note that this empirical-family ordering differs from the analytic ordering of Table 2 at the burst and long-memory extremes (see text).

Regime	1/e (Default)	1/2-Cross	Integrated	AR(1) Eff.
IID	0.954	0.954	0.977	0.977
Markov 0.7	0.856	0.892	0.877	0.899
Seasonal	0.942	0.942	0.893	0.971
Burst	0.500	0.500	0.500	0.655
Long-memory	0.954	0.954	0.977	0.977

Together these ablations show that the proxy model’s qualitative regime ordering is robust to τ estimator choice and forward-window size, while the absolute proxy values are sensitive to the constants *C* and δ (Table 8 quantifies the induced movement of the crossover $\rho^{*}$) and to the stream length *T*. The Burst regime is the most sensitive: at T=4096 it is in proxy saturation under most estimators, but emerges from saturation as *T* doubles. These findings support the paper’s claim that the (τ,r) landscape is a hypothesis-generating screening heuristic rather than a precise prediction tool.

## 7. Discussion

### 7.1. Implications for Quantum Streaming Applications

Three structural factors are consistent with a hypothesised proxy-favourable regime: (1) the paper-defined O(n) vs. $\Omega (\sqrt{N})$ illustrative memory-reference gap grows exponentially with *n*, so within the proxy visualisation even large increases in τ and *r* would not eliminate that reference at n≥20—an extrapolation beyond the tested range n≤14, not a theorem or measured trend (Section 3.3); (2) analytic proxy τ overshoots empirical τ for the mixing regimes (Figure 4; Section 7.3), so for those regimes the analytic proxy is conservative and tighter family-specific characterizations would enlarge the predicted favourable region; (3) the proxy model predicts a favourable separation at mild Markov correlation (sign-concordant paired differences, $r_{\mathrm{rb}}\geq +0.71$, with exact CIs excluding zero at ρ≤0.27 and the ρ=0.48 interval one estimator lattice step from zero) that closes at $\rho^{*}\approx 0.82$–0.88 (Table 4), a range that overlaps autocorrelation levels reported in prior measurement studies; the two real-stream cases examined here (Section 6.8) place one stream inside and one outside the hypothesised favourable region, and broader validation across additional domains remains future work. All three factors are properties of the proxy model or its illustrative references; none is a measured quantum result.

The (τ,r) landscape is a hypothesis-generating screening heuristic: researchers can place a candidate stream’s estimated (τ,r) on the map to identify the most comparable synthetic regime and determine whether deeper empirical or algorithm-level study is warranted, with the contour shape robust to multiplicative proxy rescalings.

### 7.2. Practical Implications: Stream Classes on the Landscape

Table 10 translates typical autocorrelation ranges from the measurement literature into (τ,r) coordinates and records the proxy model’s qualitative prediction. This table itself reports no new measurements; the overlays rely on published statistics [3,4,5,6,7,8,50]. The new real-stream measurements in this paper are the two NAB cases (Section 6.8): NYC Taxi falls in the favourable lower-left region of the landscape, while Machine Temperature falls in the unfavourable upper-right region where the proxy saturates. Running the full pipeline at scale—broader real-stream coverage paired with an implementable quantum oracle sketching algorithm—remains an explicit follow-on target (Section 9).

### 7.3. Why Analytic and Empirical Correlation Estimates Diverge

Experiment 4 found gaps of ≈8× (Markov) and ≈125× (long-memory) between analytic and empirical τ. These gaps are systematic, and understanding them matters for anyone using the landscape, so we give the technical explanation, its interpretation, and its implications.

#### 7.3.1. Technical Explanation

The two quantities estimate *different aspects* of temporal dependence. Each analytic proxy targets a model-level, worst-case dependence scale of the *generator*: for Markov switching it tracks the mixing time of the joint *n*-bit product chain (Θ(nlogn/(1−ρ)), simplified to n/(1−ρ)), and for long-range dependence it tracks the growth of the integrated autocorrelation of the underlying Gaussian driver ($T^{2H-1}$), which is dominated by the heavy *tail* of the power-law autocorrelation function. The empirical estimator instead measures the first 1/e crossing of the *marginal, bitwise* autocorrelation of the *binarised* stream—an instance-level, short-lag quantity. For Markov switching, each individual bit decorrelates as $\rho^{k}$, crossing 1/e at k≈1/ln(1/ρ) (≈2.8 at ρ=0.7), while the joint state of all *n* bits takes Θ(nlogn/(1−ρ)) steps to mix; both numbers are correct answers to different questions. For long-range dependence the divergence is compounded three ways: the underlying process has no finite decorrelation time at all (its autocorrelation is non-summable), so any finite empirical τ is a property of the estimator rather than of the process; binarisation shrinks the autocorrelation via the arcsine law (2/π)arcsin(γ) [53]; and the 1/e-crossing responds to the fast initial decay of the power-law autocorrelation while the analytic proxy integrates its slow tail.

#### 7.3.2. Interpretation

The divergence is partly *expected behaviour* and partly a *proxy limitation*, and the long-memory regime shows why the two cannot be fully separated: for a non-mixing process there is no single scalar that both estimators could agree on, so a single-number τ is intrinsically lossy there. It is *not* evidence of a data-generation defect: the fGn generator reproduces the intended Hurst statistics, and the gap persists under all four alternative τ estimators in Experiment 9, which shows that the estimator family, not an implementation artefact, is responsible. The practical consequence is stark and worth stating: under empirical τ the long-memory regime moves to the best tier of the landscape (its finite $\hat{\tau }$ measures only the fast head of a non-summable autocorrelation), while burst moves to the saturated floor (its measured τ·r is the largest of any regime)—the analytic and empirical conventions disagree at exactly the two extremes (Section 6.9).

#### 7.3.3. Implications

First, the empirical τ is the operationally meaningful input for placing a stream on the landscape: it reflects the short-lag effective information that a bounded-memory learner actually experiences over the measured horizon. Second, the analytic τ retains value as a conservative stress-test envelope: a stream that remains in the hypothesised favourable region even under the analytic τ is favourable with margin. Third, the *ratio* of analytic to empirical τ is itself a useful diagnostic—large ratios flag long-memory content whose risk a short-lag estimator will underestimate at longer horizons. Multi-scale refinements of τ (e.g., a family of integrated-autocorrelation estimates at several horizons) are a natural follow-on (Section 9).

### 7.4. Scope of the Diagnostic: What the Landscape Can and Cannot Predict

**Figure 9:**
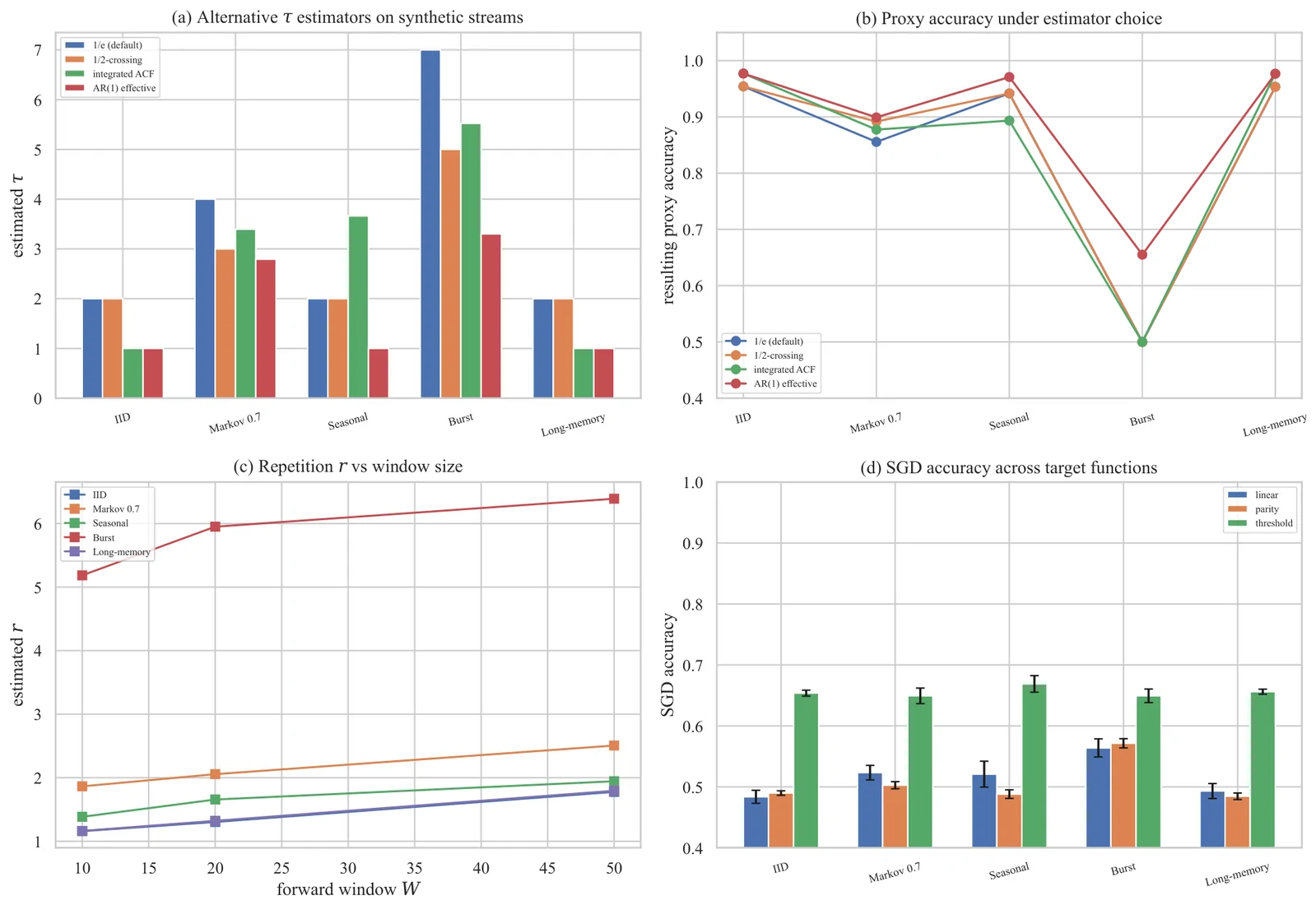
Proxy-sensitivity and estimator ablations. (**a**) Estimated τ on each regime under four alternative estimators. (**b**) Resulting proxy accuracy. (**c**) Repetition number *r* versus forward-window size *W*. (**d**) Online-SGD accuracy across three target functions (linear, parity, threshold), 10-seed mean with 95% CI. The proxy curve is target-independent by construction; it does not track task-specific learnability.

The experiments delimit the diagnostic sharply, and we state the boundary explicitly. The landscape *can*: (i) rank correlation regimes and real streams by proxy-estimated effective sample budget, using estimators that apply unchanged to synthetic and real streams (Experiments 4 and 8); (ii) flag streams whose correlation structure erodes the budget so severely that no sample-limited method should be expected to recover a rare class (Machine Temperature at $T_{\mathrm{eff}}=52$); and (iii) generate falsifiable, regime-level hypotheses about where an implemented oracle-sketching algorithm would be most worth evaluating. The landscape *cannot*: (i) predict the accuracy of a specific classifier on a specific target—classical performance is target-dependent while the proxy is target-independent (Experiment 9, Figure 9d); (ii) anticipate representation-specific effects such as the Count-Min memorisation route on coarse binarisations (Experiment 8); (iii) predict classical-learner accuracy from $T_{\mathrm{eff}}$ even on a fixed target—measured classical accuracy is flat across the Markov sweep while $T_{\mathrm{eff}}$ falls roughly fivefold (Figure 5), so the effective-sample construction is a budget model, not a performance model, even for the classical arm; or (iv) certify quantum performance, because no quantum algorithm is implemented. In short, the landscape characterises correlation structure, not task-specific learning performance, and its outputs are hypotheses for validation rather than performance guarantees.

#### Falsifiable Hypotheses

To make the hypothesis-generation claim concrete, the landscape’s headline predictions are stated with refutation criteria, each bound to the estimator convention it uses. **H1** (ordering): an implemented or simulated oracle-sketching pipeline, evaluated at matched *T* and *n* across the five synthetic regimes at their *empirical*(τ,r) coordinates, ranks the regimes in the same order as acc_proxy (Spearman $\rho_{s}\geq 0.8$); refuted if $\rho_{s}<0.5$. **H2** (budget floor): on any stream whose measured $T_{\mathrm{eff}}<100$ under the published estimators, no bounded-memory learner with M≤256 exceeds balanced accuracy 0.60 on a 20%-rare-event target; refuted by a single counterexample (Machine Temperature, $T_{\mathrm{eff}}=52$, is the first instance and is so far consistent). **H3** (crossover): under the operating constants and the empirical-τ convention, an implemented pipeline’s accuracy advantage over online SGD on the Markov family changes sign within ρ∈[0.75,0.95]; refuted if the sign change falls outside that interval or does not occur. These are proxy-level predictions; testing them is the first priority of Section 9.

## 8. Threats to Validity

We systematise the study’s limitations as threats to validity, stating for each how it is mitigated and what residual risk remains.

### 8.1. Construct Validity

The central constructs—τ, *r*, $T_{\mathrm{eff}}$, and the accuracy proxy—are operational stand-ins for theoretical quantities they do not provably equal (Section 3.3). Four specific risks follow. (i) No quantum algorithm is implemented or simulated, so “quantum” curves measure the heuristic model, not quantum computation. (ii) τ and *r* are not formally equivalent to universal mixing-time, effective-sample, or quantum-overhead quantities, and Section 7.3 shows that a single-scalar τ is intrinsically lossy for non-mixing processes. (iii) The accuracy proxy applies a task-level generalisation rate rather than the cost of an implemented quantum algorithm (Experiment 6). (iv) Feature binarisation changes the correlation structure it is meant to measure (arcsine shrinkage), so real-stream (τ,r) estimates are lower bounds on the underlying continuous-process correlation. Mitigations: the provenance of the model-level quantities is tabulated (Table 1) with family-specific notes in Section 4, and the estimator ablations (Experiment 9) establish ordering stability *within* the empirical estimator family—while exposing that the analytic and empirical conventions disagree at the burst and long-memory extremes (Section 7.3), a disagreement the text reports rather than conceals.

### 8.2. Internal Validity

(i) The headline comparisons pair a stochastic learner with a deterministic-given-$\hat{\tau }$ model prediction; they are seed-stability statements, not algorithm-versus-algorithm outcomes, and are labelled as such (Experiment 5). (ii) The same stream realisation is used both to estimate (τ,r) and to evaluate the classical baselines, which could couple estimation error with baseline noise; the narrow CI bands and the estimator ablations bound this effect. (iii) Classical hyperparameters (learning rate, hash width *M*) are fixed across regimes rather than tuned per regime; conclusions about the proxy are unaffected, but per-regime classical accuracies should not be read as the best classically achievable. (iv) The classical baselines are practical learners on a simple linear task, not the problems addressed by the cited quantum-streaming separations, so no comparison here is an algorithm-versus-algorithm benchmark of that literature. (v) On real streams, the rare-event target threshold is computed from the full series—look-ahead in the target definition, affecting the classical reference numbers only, since the (τ,r) placement is target-free—and baselines are evaluated on permuted orderings whose intervals measure ordering variability of one fixed series (Section 6.8).

### 8.3. External Validity

(i) The five synthetic families are author-chosen idealisations of documented stream phenomena; other correlation structures (regime-switching with heavy-tailed sojourns, adversarial drift) are unrepresented. (ii) Only two real-world datasets are analysed, both from NAB telemetry; the real-stream findings are illustrative, and generalisation to network telemetry, cybersecurity event streams, financial series, and large IoT fleets is unverified (Section 9). (iii) Dimensions are modest (n≤14); moreover $\tau_{\mathrm{Markov}}=n/\left(1-\rho \right)$ grows linearly in *n*, so the analytic Markov proxy becomes less favourable at higher dimensions, making family-specific refinements most valuable there. The paper-defined memory curves do not resolve this external-validity limitation. (iv) The rare-event target on real streams is a per-stream 80th-percentile threshold, not the original NAB anomaly labels, so results do not transfer to NAB benchmark comparisons.

### 8.4. Statistical-Conclusions Validity

All stochastic results use 10 seeds; marginal intervals are 1.96×SE normal approximations, while paired differences use exact signed-rank intervals (Table 3). The five sweep comparisons do not survive Holm correction at the family-wise 0.05 level; this is an empirical outcome of one-to-two discordant seeds, not a design ceiling (the smallest attainable adjusted *p* at n=10 is 0.0098), and the crossover claim accordingly rests on effect sizes and interval estimates rather than significance (Table 4). The paired tests’ interpretation is further restricted by the deterministic-vs-stochastic pairing above. Absolute landscape boundaries are conditional on the constants (C,δ) (Table 8); only ordinal statements are robust across the grid.

## 9. Future Work

The framework’s outputs are hypotheses, and the priority for future work is to test them. (i) *Algorithm-level validation*: implement or simulate an oracle-sketching pipeline at small *n* and compare its measured behaviour against the landscape’s regime-level predictions—the single step that would convert the hypotheses generated here into evidence. (ii) *Formal foundations*: derive bounds connecting the operational (τ,r) to effective information and algorithm-level behaviour, including multi-scale refinements of τ for non-mixing processes (Section 7.3). (iii) *Broader real-stream corpora*: extend the two-dataset sanity check to network telemetry (flow and latency series), cybersecurity event streams (intrusion and log-anomaly feeds), financial tick and returns series, and large-scale IoT monitoring fleets, characterising each stream’s (τ,r) and mapping it onto Figure 3 with imbalance-aware metrics throughout. (iv) *Tighter family-specific proxies* that close the analytic–empirical τ gap. (v) *Task extensions* to multiclass classification and regression, and repetition-aware oracle constructions that exploit burst structure variance-reductively rather than treating duplicates as wasted samples.

## 10. Conclusions

This paper introduced a proxy-based diagnostic and hypothesis-generation framework for correlation-sensitive streaming analysis, and applied it in a computational study spanning five structured synthetic non-IID regimes, two real telemetry streams, and a five-way ablation suite. The **primary contribution** is the (τ,r) sensitivity landscape: an operational map, built from measurable correlation proxies, on which synthetic regimes and real streams are placed with the same estimators. The **secondary contribution** is the use of that landscape for hypothesis generation: it identifies where quantum oracle sketching is most likely to remain robust, and therefore where algorithm-level validation effort should be spent first. The paper demonstrates no quantum advantage and implements no quantum algorithm; every quantum-side quantity is a heuristic proxy whose provenance and epistemic status are tabulated in Section 3.3.

### 10.1. Research Question Answers

- **RQ1**: Analytic and empirical τ estimates diverge by ≈8× (Markov) to ≈125× (long-memory); for non-mixing processes the two are not commensurable, and Section 7.3 explains the divergence as two estimators answering different questions about temporal dependence. The generic analytic proxy is qualitatively informative but quantitatively loose.
- **RQ2**: The (τ,r) landscape (Figure 3) organises the regimes and both real streams; its ordering is stable *within* the empirical estimator family and across window sizes and proxy constants (Experiment 9), while the analytic and empirical conventions disagree at the burst and long-memory extremes and absolute boundaries are conditional on (C,δ) (Table 8). τ and *r* are effective-sample-budget summaries, not accuracy predictors (Figure 9d).
- **RQ3**: Under the *analytic* proxies the hypothesised favourable region covers IID and burst, and the proxy-predicted separation vanishes in the long-memory regime; under the *empirically measured* (τ,r) those two extremes exchange places (Section 6.9)—an estimator dependence that is itself a finding. On the Markov family, characterised under both conventions, the paired proxy − classical difference declines from +0.042 to +0.019 (Hodges–Lehmann) and reverses sign (−0.046, exact CI excluding zero), with the mean curves crossing between ρ≈0.81 and 0.88 under the operating constants; this closure is the robust finding.
- **RQ4**: Analytic *r* and empirical *r* agree within 1.4× for burst and are near-identical for all other regimes under unit-aligned measurement; the directional τ gaps are monotone in correlation strength and admit family-specific refinement.

### 10.2. Practical Contribution

For stream-processing practitioners, the framework is a cheap pre-screening step: estimate (τ,r) with the paper’s estimators, place the stream on the landscape, and read off whether correlation alone already erodes the effective sample budget to a level at which rare-event learning is unlikely—as it does for the Machine Temperature stream ($T_{\mathrm{eff}}=52$)—before investing in any advanced pipeline, quantum or classical. The real-stream check (Experiment 8) shows the workflow runs end-to-end on public telemetry, with imbalance-aware metrics guarding against majority-class artefacts.

### 10.3. Limitations and Required Validation

The framework’s outputs are hypotheses conditioned on a heuristic model; Section 8 systematises the construct, internal, external, and statistical threats. The decisive missing step is algorithm-level validation: an implemented or simulated oracle-sketching pipeline evaluated at the landscape’s predicted-favourable and predicted-unfavourable coordinates (Section 9). Until that step is taken, the landscape should be used as a prioritisation tool, not as evidence of quantum benefit.

## Figures and Tables

**Table 1:** Provenance and epistemic status of the model-level quantities used in this paper; the family-specific proxy expressions (Equations (5)–(8)) carry their own provenance notes in Section 4. “Published background” items are supported by the cited literature; “introduced here” items are operational constructions of this paper; and the final column states what is *not* theoretically guaranteed.

Quantity	Status	Not Guaranteed
O(n) quantum and $\Omega (\sqrt{N})$ classical memory curves	introduced here (illustrative scaling reference)	a proven separation for this diagnostic task
τ (1/e ACF crossing)	introduced here (operational)	universal equivalence to an effective-sample or mixing time
*r* (*W*-window duplicate count)	introduced here (operational)	a quantum sample-overhead theorem
$T_{\mathrm{eff}}=T/\left(\tau r\right)$	introduced here (conservative)	correspondence to any exact sample-complexity theorem
$\epsilon \leq C\sqrt{n\log\left(1/\delta \right)/T_{\mathrm{eff}}}$	standard VC-type task rate, repurposed here	equality with any quantum-algorithm cost
$\mathrm{acc}\_\mathrm{proxy}=\max(0.5,\;1-\epsilon^{2})$	introduced here (visualisation surrogate)	correspondence to any implemented algorithm’s accuracy
$\Omega (\sqrt{N})$ memory curve	introduced here (illustrative scaling reference)	a classical lower bound for this diagnostic task

**Table 2:** Summary of heuristic correlation proxies and MSE proxy values ($\epsilon^{2}$) for each regime, with T=10,000, n=10, C=2, and δ=0.05. The factor $\kappa =C^{2}\log\left(1/\delta \right)\approx 12$.

Regime	τ	*r*	$T_{\mathrm{eff}}$	MSE Proxy
IID	1	1	10,000	0.012
Markov (ρ=0.7)	n/(1−ρ)≈33	1	≈300	0.40
Seasonal (P=100,α=0.3)	max(1,Pα)=30	1	≈333	0.36
Burst (L=10,p=0.3)	1	1+pL=4	2500	0.048
Long-memory (H=0.8)	$T^{2H-1}\approx 251$	1	≈40	1.0 (saturated)

**Table 3:** Reproducibility summary: fixed experimental parameters.

Parameter	Value/Method
Seeds	10/experiment (42–51, Exps. 1–7; 0–9, Exps. 8–9)
Forward window *W*	20
CI construction	marginal: ±1.96SE; paired: exact signed-rank
Proxy constants	C=2, δ=0.05, $\kappa =C^{2}\log\left(1/\delta \right)\approx 12$
$\tau_{\mathrm{emp}}$ estimator	smallest lag *k* with mean bitwise ACF <1/e
$r_{\mathrm{emp}}$ estimator	1 + mean exact-duplicate count in *W*-forward window
Wilcoxon pairing	per-seed classical accuracy vs. same-seed proxy value
Stochastic quantities	classical accuracy, $\tau_{\mathrm{emp}}$, $r_{\mathrm{emp}}$ (10-seed, 95% CI)
Deterministic quantities	$T_{\mathrm{eff}}$, $\epsilon^{2}$, acc_proxy, (τ,r) landscape

**Table 7:** Encoding/resolution sensitivity of the real-stream placements (deterministic re-analysis of the same raw series; n=6, W=20, C=2, δ=0.05). The NYC Taxi > Machine Temperature proxy ordering holds in all ten configurations; absolute values are encoder-conditional.

Stream	Resolution/ToD Bucket	*T*	τ	*r*	$T_{\mathrm{eff}}$	Proxy
NYC Taxi	30 min/3 h (published)	10,320	6.0	2.77	621	0.884
NYC Taxi	30 min/6 h	10,320	8.0	4.55	283	0.746
NYC Taxi	30 min/1.5 h	10,320	4.0	1.89	1367	0.947
NYC Taxi	60 min/3 h	5160	4.0	1.64	785	0.908
Machine Temp.	5 min/3 h (published)	22,695	32.0	13.55	52	0.500
Machine Temp.	5 min/6 h	22,695	52.0	16.04	27	0.500
Machine Temp.	5 min/1.5 h	22,695	22.0	9.32	111	0.500
Machine Temp.	30 min/3 h	3781	7.0	3.18	170	0.576
Machine Temp.	30 min/6 h	3781	10.0	5.59	68	0.500
Machine Temp.	60 min/3 h	1891	4.0	1.85	255	0.719

**Table 10:** Qualitative mapping of real stream classes onto the (τ,r) landscape (Figure 3c). Reported parameter ranges are from the cited measurement literature; all placements use the *analytic*-τ convention and the paper’s proxy formula at $T=10^{4}$, n=10. The final column records hypotheses generated by the proxy landscape for future algorithm-level testing, not demonstrated performance.

Stream Class	Reported Range	Landscape Region	Proxy Hypothesis
Network backbone	H≈0.75–0.95	upper-right (long-memory)	*Unfavourable*
Financial returns	H≈0.55–0.70	τ≈2.5–40	*Favourable (H≲0.6) → borderline*
Sensor/IoT	burst + mid-ρ Markov	0.6–0.8 contour band	*Borderline*
Concept-drifted	seasonal, moderate drift	≈0.6–0.65 contour	*Modest at best*

## Data Availability

The two real-world datasets analysed in this work—NYC Taxi pickup counts and machine-temperature sensor readings—are publicly available under the MIT licence in the Numenta Anomaly Benchmark repository [54]. All other data are synthetic and generated by seeded random-number generators with the parameters specified in Section 5 and Table 3; every stochastic experiment uses 10 seeds (indices 42–51 for the synthetic suite, 0–9 for the ablation and real-stream harnesses). A reference Python implementation of the stream generators, classical baselines, proxy formulas, real-stream loader, and ablation harness is openly available under the MIT licence and archived at Zenodo (DOI https://doi.org/10.5281/zenodo.19831893) (accessed on 16 August 2026), with an active mirror on GitHub (https://github.com/mmahmoudai/Beyond-IID-Data-A-Proxy-Based-Computational-Study-of-Quantum-Oracle-Sketchin) (accessed on 16 August 2026). The repository regenerates all figures and the measured tables; the effect-size, constant-sensitivity, and encoder-sensitivity tables are deterministic re-analyses of those outputs whose scripts are included in the revision package.
